# Comparative analysis of Constitutive and fiber-specific promoters under the expression pattern of Expansin gene in transgenic Cotton

**DOI:** 10.1371/journal.pone.0230519

**Published:** 2020-03-18

**Authors:** Amina Yaqoob, Ahmad Ali Shahid, Ibrahim Bala Salisu, Sana Shakoor, Muhammad Usmaan, Mohsin Shad, Abdul Qayyum Rao

**Affiliations:** Centre of Excellence in Molecular Biology, University of the Punjab, Lahore, Pakistan; Nigde Omer Halisdemir University, TURKEY

## Abstract

Promoters are specified segments of DNA that lead to the initiation of transcription of a specific gene. The designing of a gene cassette for plant transformation is significantly dependent upon the specificity of a promoter. Constitutive Cauliflower mosaic virus promoter, *CaMV35S*, due to its developmental role, is the most commonly used promoter in plant transformation. While *Gossypium hirsutum* (*Gh*) being fiber-specific promoter (*GhSCFP*) specifically activates transcription in seed coat and fiber associated genes. The Expansin genes are renowned for their versatile roles in plant growth. The overexpression of Expansin genes has been reported to enhance fiber length and fineness. Thus, in this study, a local Cotton variety was transformed with Expansin (*CpEXPA1*) gene, in the form of two separate cassettes, each with a different promoter, named as *35SEXPA1* and *FSEXPA1* expressed under *CaMV35S* and *GhSCFP* promoters respectively. Integration and Spatiotemporal relative expression of the transgene were studied in an advanced generation. *GhSCFP* bearing transgene expression was significantly higher in Cotton fiber than other plant parts. While transgene with *CaMV35S* promoter was found to be continually expressing in all tissues but the expression was lower in fiber than that expressed under *GhSCFP*. The temporal expression profile was quite interesting with a gradual increasing pattern of both constructs from 1DPA (days post anthesis) to 18DPA and decreased expression from 24 to 30 DPA. Besides the relative expression of promoters, fiber cellulose quantification and fluorescence intensity were also observed. The study significantly compared the two most commonly used promoters and it is deduced from the results that the *GhSCFP* promoter could be used more efficiently in fiber when compared with *CaMV35S* which being constitutive in nature preferred for expression in all parts of the plant.

## 1. Introduction

The cotton is the backbone of the global textile industry. More than fifty-five countries are dealing in cotton cultivation, fiber processing, and export. Pakistan is the fourth-largest consumer and exporter of Cotton [1]. It comes in the fifth position among the largest Cotton producing countries of world and third position for having the largest capacity of raw fiber spinning in the world [2]. Several efforts have been made to improve the fiber quality and yield to strengthen up this industry and increase the fiber export. Fiber is an epidermal single-cell extension of a plant seed. Pure fiber can be isolated during sequential stages of differentiation i.e. primary and secondary wall synthesis/thickening [3]. Fiber development is divided into three overlapping phases of Fiber initiation, elongation, and maturation. The fiber cell wall elongation phase is characterized by unique, cell wall loosening, non-enzymatic proteins called Expansins [4]. Expansins are meant for cell wall expansion or elongation and naturally express in all plants [5, 6]. Qualitative and quantitative traits of Cotton fiber are significantly dependent on the cell wall development and expression pattern of cell wall synthesis genes [7]. Cell wall-related transcription factors and several genes like Expansin, cellulose synthase, Sucrose synthase, and Actin have been successfully transformed into Cotton with improved fiber, enhanced qualitative and quantitative characteristics [8]. Expansin is one of the most important fiber promoting genes which not only affects the fiber elongation but also the cellulose deposition. Besides genes, one cannot ignore the role of promoters in transformation studies [9]. A number of attempts have been made so far to transform cotton with the Expansin gene for improving fiber qualities, but the selection of an appropriate promoter has not been focused yet.

Promoters are primarily divided into three types, (1) Constitutive, (2) Spatiotemporal and (3) inducible. The most commonly used promoter is constitutive type, *CaMV35S*, which allows the continuous gene expression in a stable manner [10]. However, Spatiotemporal promoters, being specific in their expression for a particular gene under particular conditions, are predominating the constitutive ones. *GhSCFSP* is an example of a Spatiotemporal promoter [11]. The inducible promoters remain inactive until they are stimulated by the application of an external signal. Primarily, all these promoters have similar core sequences i.e. TAT-box, initiator and cis-acting motifs where binding of transcriptional factors takes place. Plant cell promoters are almost similar even in their expression but dependent upon the availability of binding transcriptional factors [12]. Transcriptional factors for the constitutive type of promoters are active and available all the time, increasing the chances of their continual expression throughout the plant organs. Whereas, those factors which bind to the other two types of promoters are limited and restricted to particular conditions or responses, therefore allow a selective promoter expression [13]. Keeping in view, the significance of promoter and Expansin gene in fiber development, we design the present study to compare the relative expression of both promoters under the expression of the Expansin gene in Cotton through Agrobacterium-mediated transformation. Positive transgenic plants from transgenic generation # 1 lines (T1 generation) were selected for this purpose.

## 2. Materials and methods

All the work is conducted at Center of Excellence in Molecular Biology (CEMB), University of the Punjab under controlled conditions.

### 2.1. Plasmid construction and cloning

**Fig 1:**
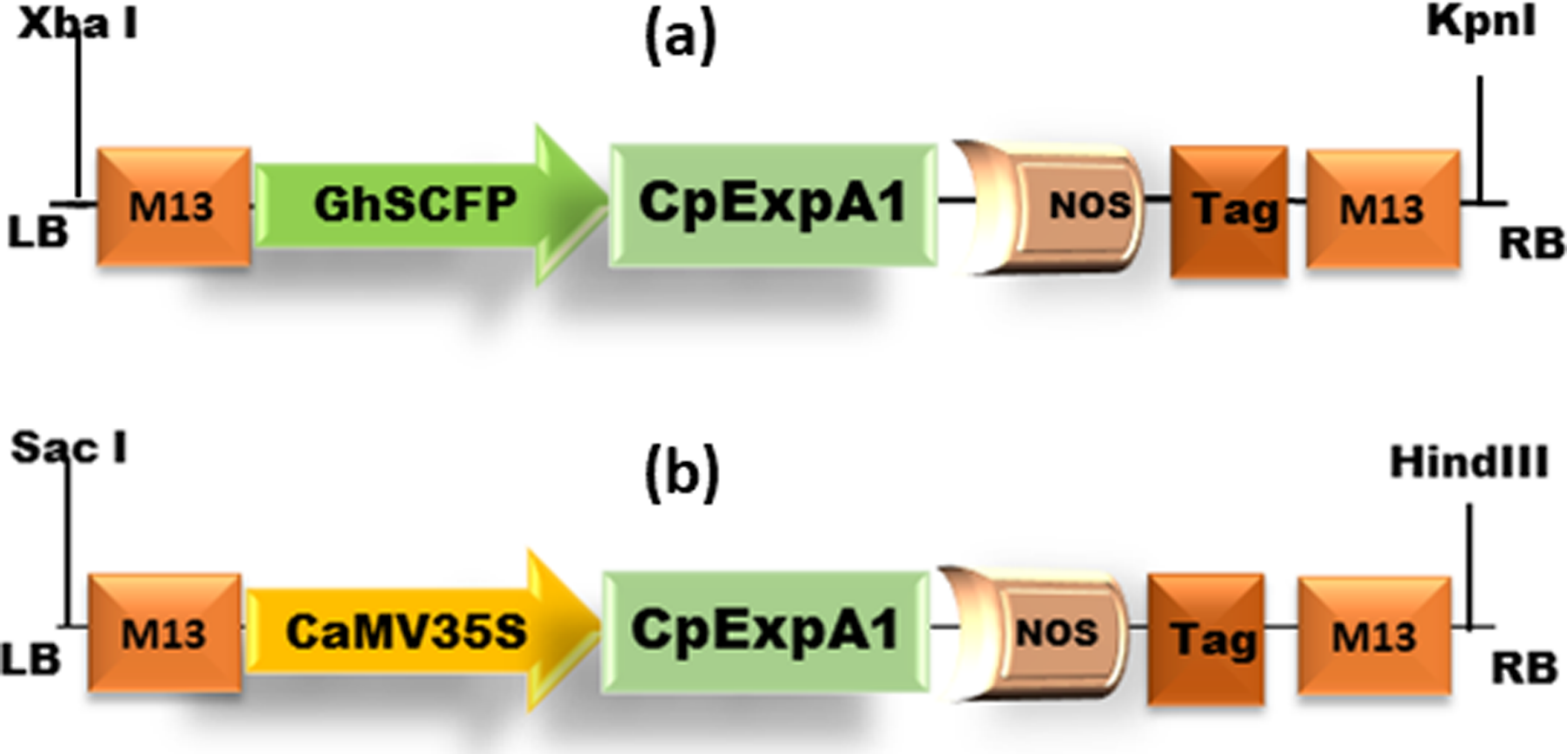
Schematic representation of *CpEXPA1* expression vectors. (a) *FSEXPA1* and (b) *35SEXPA1*. *GhSCFP* promoter: *Gossypium hirsutum* Seed coat and Fiber specific protease promoter; *CpEXPA1* gene: *Calotropis procera* Expansin-like A1; Tag: gene-specific tag; NOS: NOS terminator.

The whole nucleotide sequences of *Calotropis procera* expansin-like A1 *(CpEXPA1)* were retrieved from NCBI (https://www.ncbi.nlm.nih.gov/) with accession number, *GenBank*: *EF434781*.*2*. The in-silico joining of NCBI retrieved Expansin-like A1 gene sequence with two different promoters was done i.e. constitutive Cauliflower Mosaic Virus, *CamV35S*, [14] and *Gossypium hirsutum* seed coat Fiber-specific protease promoter, *GhSCFSP*, [15] with final gene constructs as *35SEXPA1* and *FSEXPA1* respectively (Fig 1A and 1B). Protein translation, Codon optimization, functional protein synthesis, and restriction site analysis were done in silico through online tools of Expert Protein Analysis System (ExPASy) [16], Integrated DNA Technology website [17], ExPASy and WEB-CUTTER 2.0 [18] respectively. The synthesized constructs were cloned into the binary vector of p-Cambia 1302 (Fig 1) which specifically possesses Green fluorescent protein (GFP) as a detection signal under ultraviolet light (UV) [19].

### 2.2. Plant transformation and GFP fluorescence

An approved local *Gossypium hirsutum* variety, CEMB-066, was obtained from the CEMB research repository, University of the Punjab, Pakistan, and used for all transformation experiments. Cotton seeds were transformed with Agrobacterium-mediated transformation by the shoot apex cut method as reported by [20]. An empty vector without any transgene was also transformed into the same variety as a mock transformation. Following acclimatization, the putative transgenic and control plants were shifted to the field and subjected to molecular analysis afterward. However, the presence of a transformed vector was confirmed at first in transgenic plants. Thin random sections of Cotton boll and leaf samples were taken from control (empty vector) and independent transgenic lines (*FSEXPA1 and 35SEXPA1*). The samples were sliced in distilled water and fixed in 4% Paraformaldehyde 1% glutaraldehyde solution. Fixed tissue slides were observed for GFP fluorescence using Fluorescent microscope Olympus IX83, Olympus cell sense tool, at 20X under UV light of 488nm excitation and 509nm fluorescence emission. GFP fluorescence confirmed the presence of a transformed vector.

### 2.3. Confirmation of *FSEXPA1* and *35SEXPA1* genes in putative transgenic plants

The emerging leaves from both transgenic and control plants were taken and ground in Liquid Nitrogen. CTAB method of manual DNA extraction was used [21]. The extracted genomic DNA was confirmed for the presence of transgenes by a conventional polymerase chain reaction, PCR, using primers designed for respective sequences. The pairs of primers were used for transgenes detection in plants. The PCR conditions were initial denaturation at 95C for 5 min; 30 cycles of denaturation at 95C for 30 seconds, annealing at 63.5C for 30 sec (*35SEXPA1* gene) and 59.9C (*FSEXPA1* gene), extension at 72C for 60 sec and a final extension at 72C for 10 minutes. The presence and stable integration of both transgenes, *35SEXPA1* and *FSEXPA1*, in genomic DNA, was confirmed through Dot Blot analysis as described by [21] and Fluorescent In Situ Hybridization (FISH) as described [22, 21].

### 2.4. Transgene relative expression analysis through quantitative real-time PCR

RNA extraction of different plant tissues and bolls was done as reported by [23]. Boll samples were collected from five independent transgenic lines each in triplicate, as well as from the control line, at five regular intervals (1, 6, 12, 18, 24 and 30 DPA). From each of the transgenic lines, four plant tissues (in triplicate) were selected for relative spatial expression analysis i.e. (1) newly formed leaves, (2) Cotton flower petal, (3) Immature Boll and (4) Sepals. The complementary DNA (cDNA) was synthesized using reverse transcriptase PCR (rt-PCR) (Maxima SYBR Green/ROX qPCR Master Mix (2X), Catalog # K0221, Thermo Scientific, USA). Oligo (dt) primers were used for the conversion of mRNA transcripts into cDNAs. The cDNA of *FSEXPA1* and *35SEXPA1* genes were exponentially amplified with PCR using gene-specific primers. The concentration of the amplicon was monitored with SYBR Green dye. The endogenous expression of glyceraldehyde 3- phosphate dehydrogenase (GAPDH) was used as an internal control (Table 1). All PCR reactions were performed in triplicate, with the following conditions: 95°C for 5 min and 35 cycles of at 95°C denaturation for 30 sec, annealing at 60°C (*FSEXPA1)* and 59°C (*35SEXPA1)* for 30 sec, 72°C extension for 30 sec. Each Qrt-PCR sample had three replicates [24]. For data normalization, a housekeeping gene (GAPDH) was used as a reference control, and the relative genes expression was calculated using 2^-ΔΔCT^ method described by [25].

**Table 1:** GAPDH primer sequence and accession number.

Gene name	5’-3’ sequence	Tm	Ps	Accession number
GAPDH	F-AGGAAGAGCTGCTTCGTTCA R- CCGCCTTAATAGCAGCAGCTTTG	60°C	106	XM_017782884.1

### 2.5. Cellulose quantification and fluorescence

Pure mature fiber samples (50 DPA Boll) were obtained from control and five transgenic lines of both constructs (*FSEXPA1* and *35SEXPA1*) and used for cellulose assays. For measurement of cellulose fluorescence intensity, 0.001 g of fiber sample was dried and stained with cellulose specific fluorescent dye, 0.03% Direct red (Sigma-Aldrich DR 23 212490). The stained fiber threads were thoroughly washed with water and then observed under Zeiss fluorescent microscope Axio Imager.M2, objective EC-Plan Neofluar 20X/0.30 M27, axiom 506 camera. The cellulose microfibrils retain the dye even after washing. The Fiji tool was used to measure the mean relative fluorescence intensity units (RFU). For direct quantification of cellulose, fiber samples were dissolved in acetic-acid and anthrone reagent at 65°C overnight. The pure cellulose from Avicel pH-101 (Sigma Avicel Cat# 11365-1KG) was used as a standard control to draw a standard curve. Quantification of cellulose through spectrophotometry at a wavelength of 630-635nm was done according to protocol explained by [26].

### 2.6. Statistical analysis

Graph-pad prism (version 7.0) was used for all the analyses. The values presented in the table and figures are means plus standard deviation(mean ± STD). Analysis of variance was used for data analysis. Turkey’s multiple comparisons (where applicable) was used to find any significant differences among the variables. Significant differences were considered at p<0.05.

## 3. Results

### 3.1. GFP fluorescence and transgene confirmation in transgenic plants

**Fig 2:**
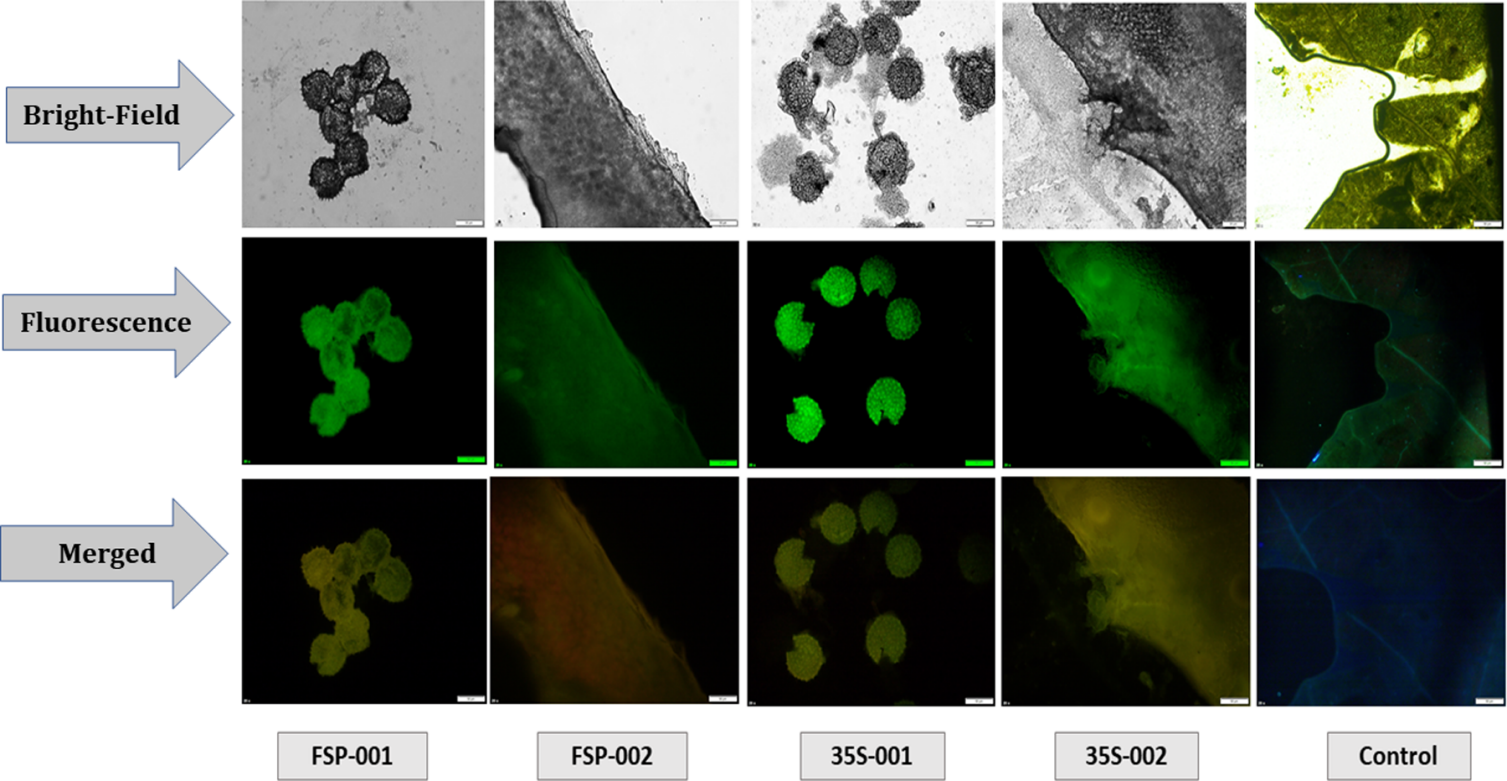
The GFP localization of *FSEXPA1*, *35SEXPA1*, and control (empty vector). The green fluorescence in different boll and leaf tissues was observed under UV. FSP-001 to 002 represents *FSEXPA1* independent transgenic lines. 35S-001 to 002 represent *35SEXPA1* independent transgenic lines. (Scale bar = 50μm).

**Fig 3:**
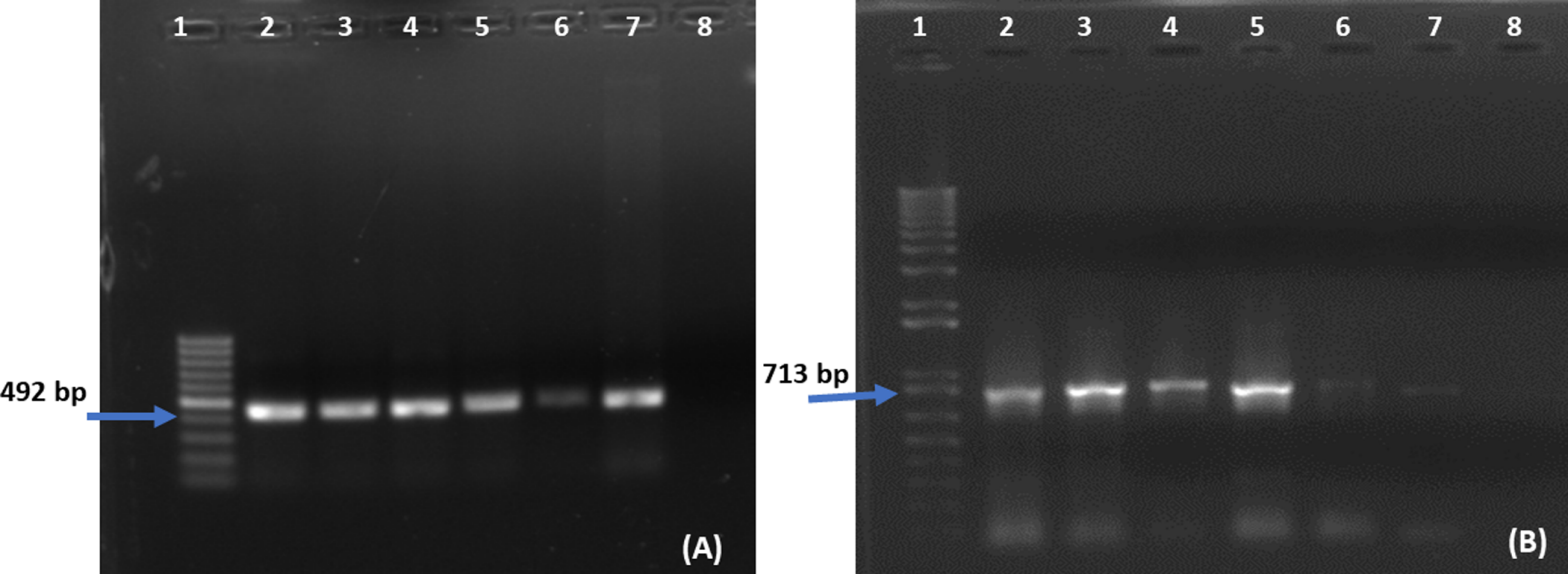
PCR amplification of *CpEXPA1* gene constructs in T_1_ transgenic plants. (A) Lane 1: 100 bp ladder, Lane 2: *35SEXPA1* Positive control, Lane 3 to 7: transgenic plants, Lane 8: (Empty vector) control (B) Lane 1: 1kb ladder, Lane 2: *FSEXPA1* Positive control, Lane 3 to 7: transgenic plants, Lane 8: (Empty vector) control.

**Fig 4:**
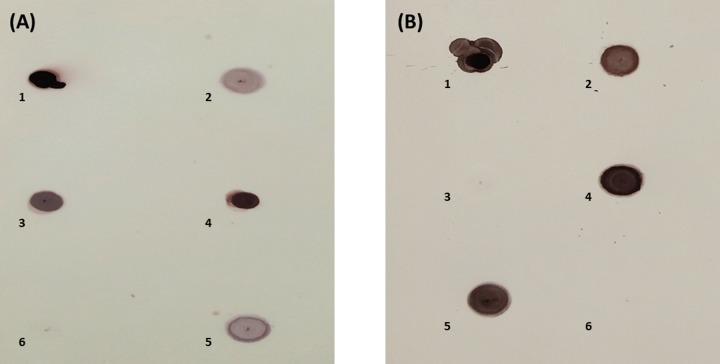
Chromogenic visualization of dot blot assay. (a) *35SEXPA1* transgenic plants, Spot 1: Positive control, Spot 2 to 5: Transgenic plants, Spot 6: Empty vector plant as Negative control (b) *FSEXPA1* transgenic plants, Spot 1: Positive control, Spot 2 to 8: Transgenic plants, Spot 9: Empty vector plant as Negative control.

GFP fluorescence was observed under UV and images were captured under bright and fluorescent fields at 20X. GFP fluorescent images confirmed the presence of a binary vector in transgenic plants (Fig 2). While the transgene presence was confirmed by PCR (Fig 3) and dot blot assay (Fig 4). The best positive plants, five plants from each transgenic group, were selected out for further analysis.

### 3.2. Fluorescent In-Situ Hybridization (FISH)

**Fig 5:**
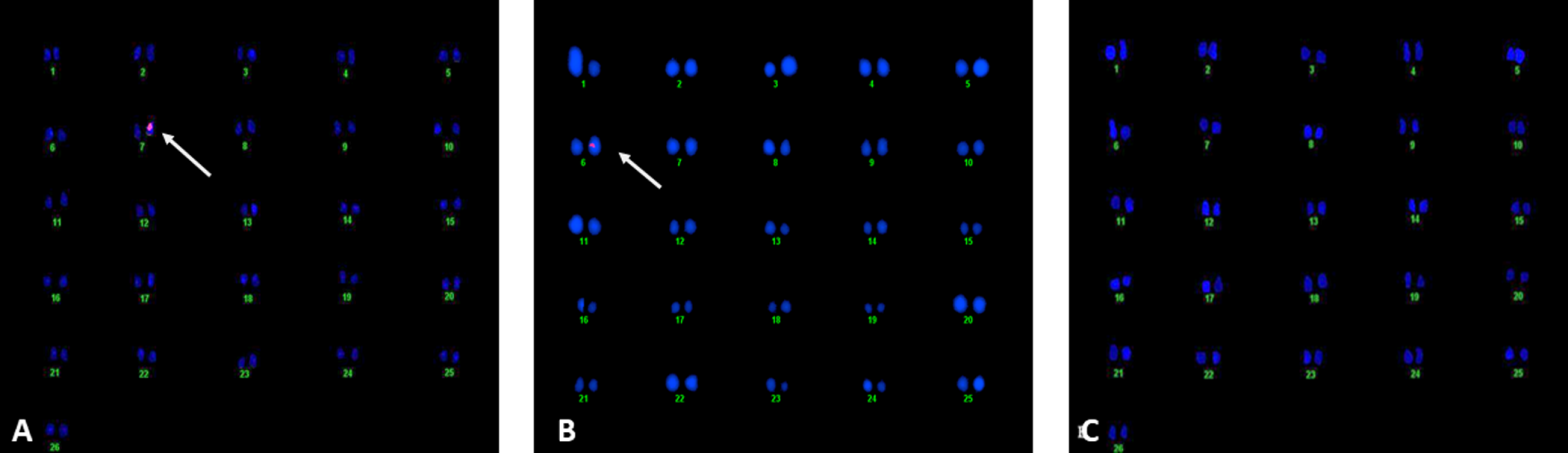
FISH analysis of transgenic embryos. (A) *FSEXPA1*, single copy number at position 7 and (B) *35SEXPA1*, single copy number at position 6 (C) Empty vector Control.

Integration and copy number of the transgene was confirmed through FISH analysis (Fig 5). The single-copy number of the transgenes in hemizygous form was detected in both transgenic cotton plants at chromosomal positions 6 and 7.

### 3.3. Cellulose quantification and fluorescence

**Fig 6:**
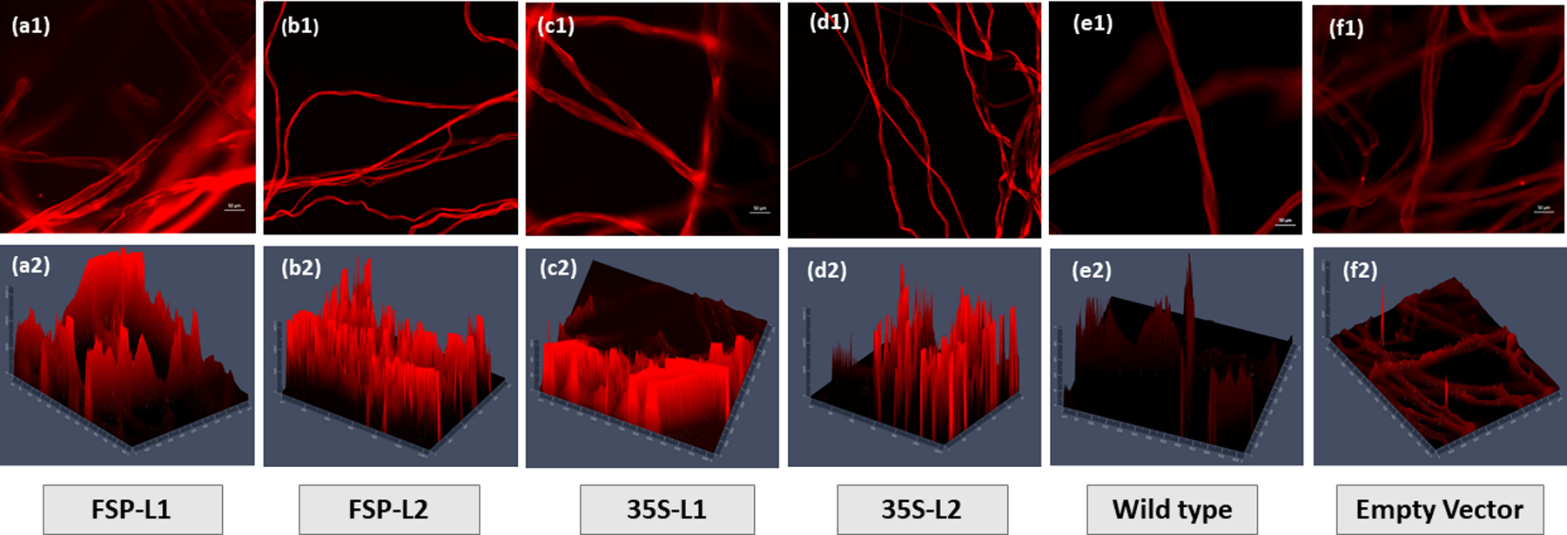
Direct red fluorescent microscopy of mature Cotton samples from transgenic and control plant lines (at 20X). The relative cellulose fluorescence in *FSEXPA1*, *35SEXPA1*, Empty vector, and Wild type. FSP-L1 and L2 represent *FSEXPA1* independent transgenic lines. 35S-L1 and L2 represent *35SEXPA1* independent transgenic lines (a1) to (f1) represent 2-dimensional images while (a2) to (f2) represent 3-dimensional images of the same line.

**Fig 7:**
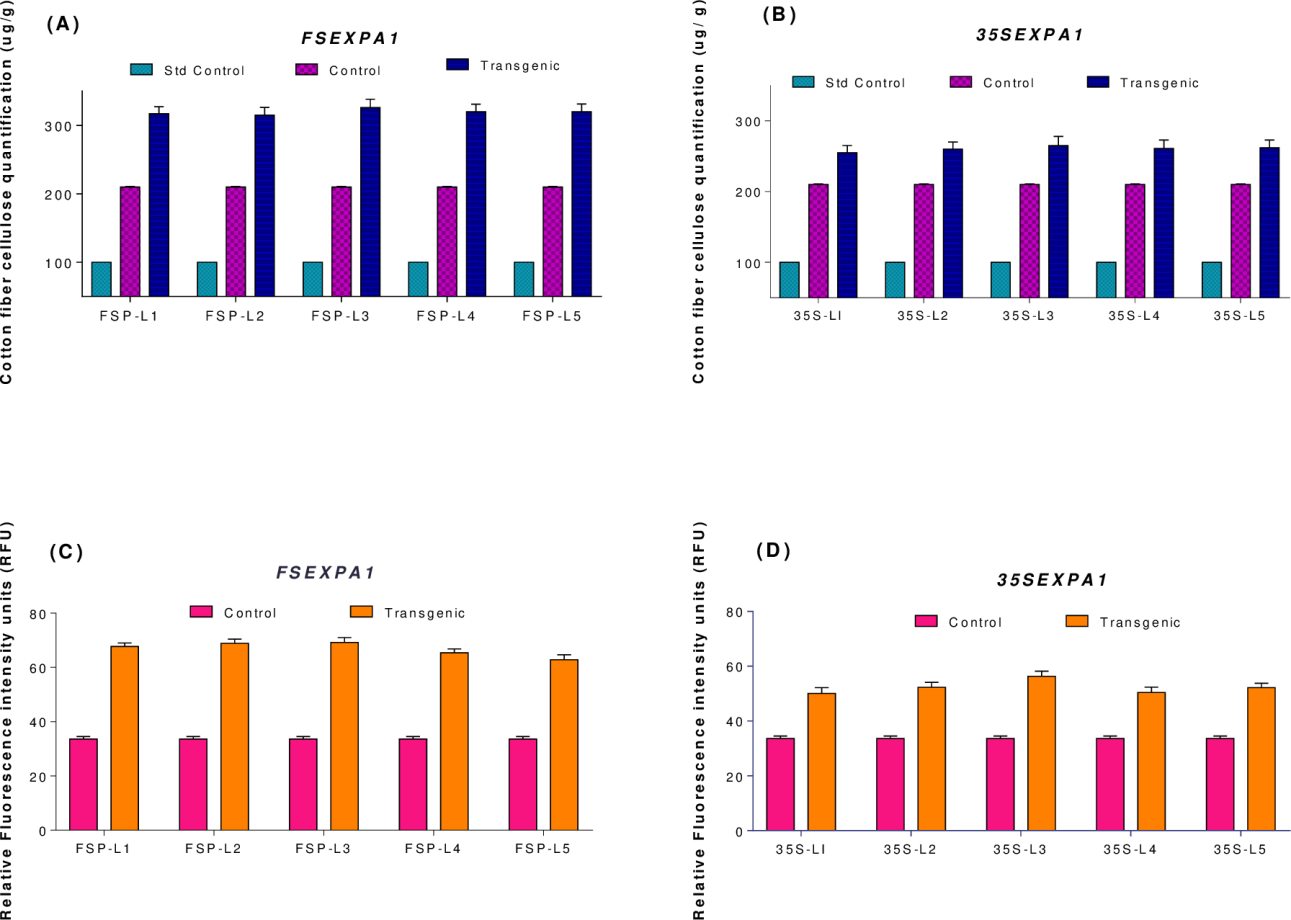
Cellulose measurement in mature fiber samples. (a, b) Cellulose quantification results and (c, d) relative fluorescence intensity values. Std; represents standard control (Avicel PH-101 pure cellulose), WT is non-transgenic wild type control, L1 to L5 represents the transgenic plant lines in all the graphs. (n = 3).

Direct Red stained fiber samples were observed under the fluorescent microscope and the result of these observations was presented as mean values of relative intensities depicted in (Fig 6). The direct red staining images were taken at 20X and analyzed in 2,5 dimensions with measurements of fluorescence intensities using the Fiji tool [27]. Five replicates of each group were examined, and the mean values of fluorescence intensities were measured. Fluorescence intensity is considered to be directly proportional to cellulose content in each fiber as direct red dye specifically stain only cellulose fibrils. The results of intensities were significantly correlated with cellulose assay quantification which confirms the authenticity of intensity values. Higher cellulose quantities and fluorescence intensities were observed in transgenic lines when compared with those in the control line (Fig 7).

### 3.4. Relative temporal and spatial expression analysis of transgenes

**Fig 8:**
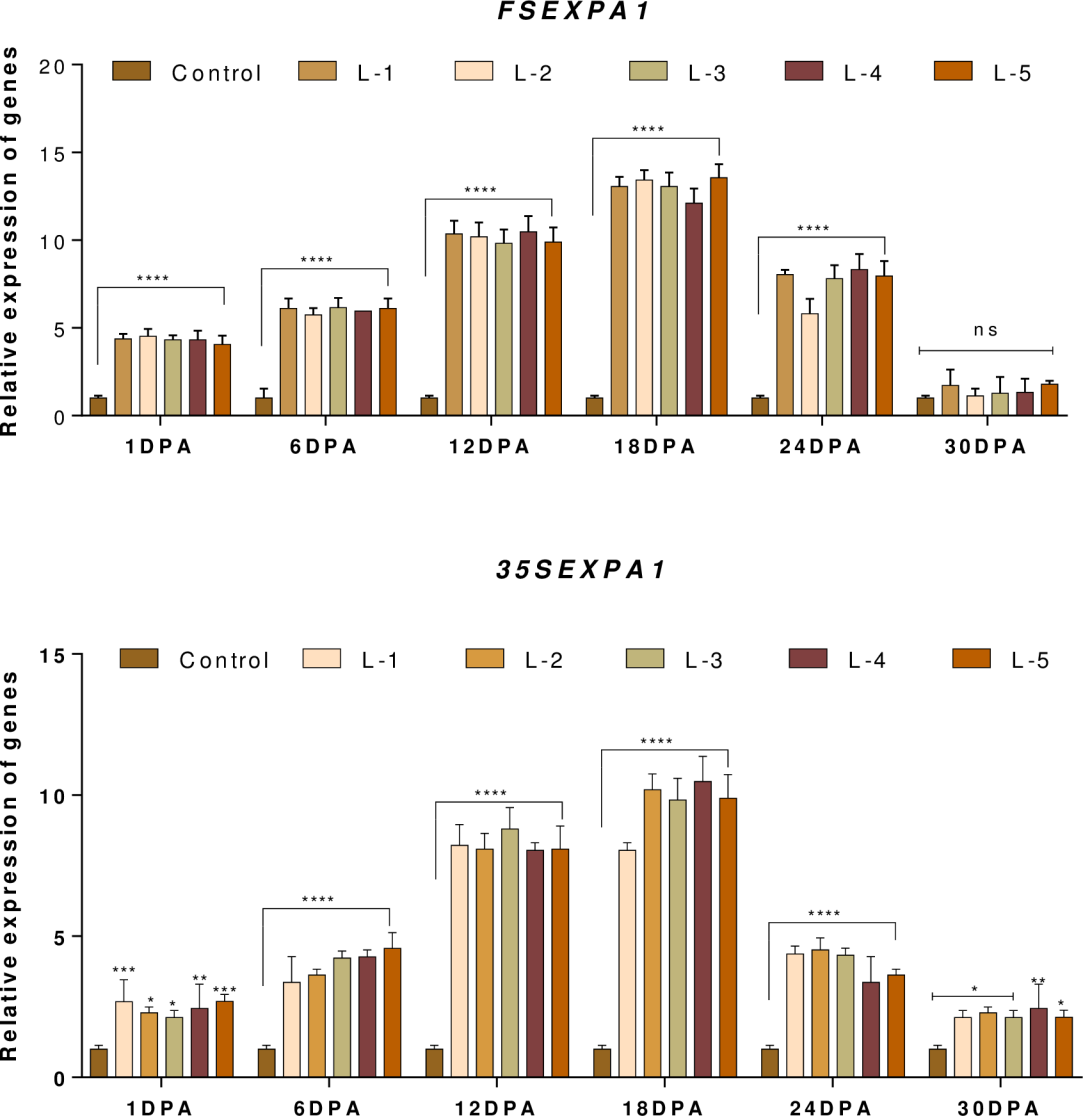
Relative expression pattern of *FSEXPA1* and *35SEXPA1* promoters in boll samples obtained from both transgenic and control lines, at regular time intervals of days post-anthesis (DPA). L1 to L-5 represent independent transgenic lines. (n = 3), where n represents both biological and technical replicates of each line.

**Fig 9:**
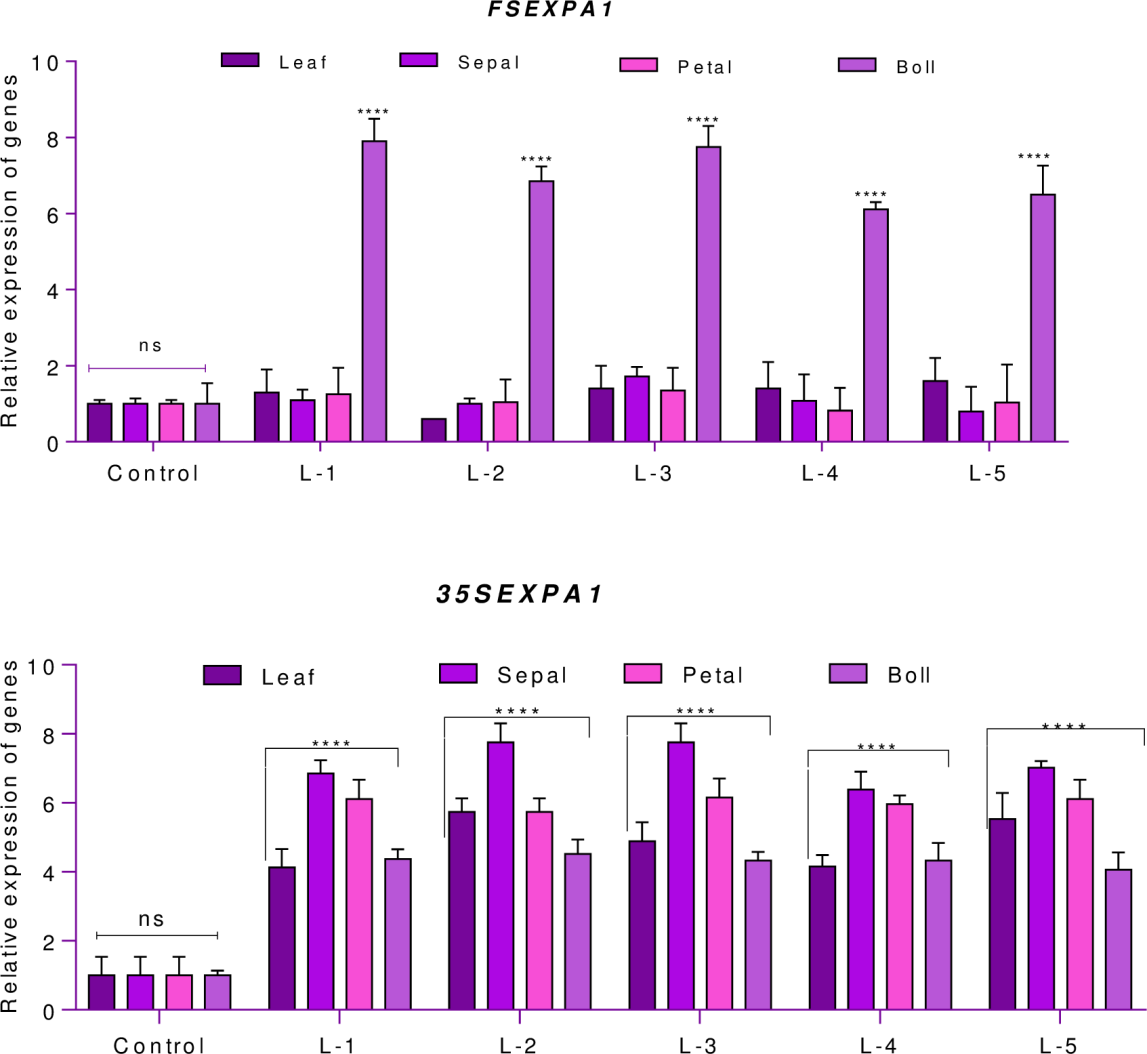
Relative expression pattern of *FSEXPA1* and *35SEXPA1* promoters in plant tissues obtained from both transgenic and control lines. L1 to L-5 represents independent transgenic lines, (n = 3), where n represents both biological and technical replicates of each line.

The *FSEXPA1* expresses spatially in bolls during 6–18 DPA. However, the gradual increase in the Expansin gene under both promoters, with higher expression under *GhSCFSP*, can be seen in (Fig 8). On the other hand, spatial expression clearly showed the specificity of *GhSCFSP* for Cotton bolls and continual expression of *CaMV35S* for all plant tissues with relatively higher expression in sepals (Fig 9).

## 4. Discussions

Cotton is one of the most important cash crops in the world economy. It is cultivated majorly for its fiber, which is the substantial raw material of the textile industry [2, 28]. Improvement in fiber quality and yield has been a center of biotechnology research and a number of experimental approaches have made so far. For this purpose, molecular approaches like overexpression, mutation, or gene silencing of fiber promoting genes, have been used [28]. Expansin, being a cell wall expansion protein, is renowned for its versatile role in fiber development. Expansin is naturally present in all plants and plays a significant role in growth and development [5, 29]. We studied the expression pattern of Expansin like-A1 gene, *CpEXPA1*, isolated from *Calotropis procera*, under constitutive and fiber-specific promoters in transgenic Cotton. The gene constructs were designed in-silico, chemically synthesized and transformed into Cotton. The T_1_ generations of both transgenic groups were studied. In transgenic technology, a green fluorescent protein (GFP) has been used widely for the confirmation of the transgenic insert in the transformed plants [30]. Similarly, in this study, the presence of a transformed vector was also confirmed by observing GFP fluorescence in transgenic plant tissues under UV (Fig 2).

The putative transgenic cotton plants were screened through PCR and Dot Blot assay. The FISH assay with gene-specific probes allows an accurate and rapid detection of copy number of respective gene sequence in dividing daughter nuclei. However, it is limited to the analysis of only one or two genes/loci at a time. The transgenic embryo meristem used to prepare the slide smear for FISH assay to target the specific transgene sequence, using respective labelled probes. A single copy number was found for both constructs (Fig 5). Correspondingly, Yasmeen et al., (2016) and Ahmad et al., (2017) also reported single transgene insertion when transgenic plants were subjected to FISH analysis [22, 31]. Moreover, the cellulose quantity and relative fluorescence intensity were also measured to assess the effect of overexpressing *CpEXA1* gene on fiber quality. *FSEXPA1* transgenic plants were found to be possessing higher cellulose in their fiber than that of *35SEXPA1* and control samples (Figs 6 and 7). These results are in accordance with the previously reported data where the Expansin gene was found to significantly increase the cellulose quantity in transgenic fiber. Cellulose deposition has a direct correlation with Expansin gene expression as cell wall loosening and tightening during the elongation phase, creating slipping of cellulose fibrils and eventually creates enough room for cellulose deposition [28, 32, 33]. An increase in cellulose content of transgenic varieties can be linked with the cell wall extension promoted by the *CpEXPA1* gene, which could have been resulted in creating more space for cellulose deposition during the fiber maturation phase [29].

Giving an insight into previously reported specificity of *GhSCFP* [15, 34, 35], the expression pattern of *CpEXPA1* under *GhSCFP* promoter was found to be higher in Cotton boll than that of the *CaMV35S*. The *GhSCFP*, isolated from Cotton, when allowed to express in Arabidopsis, Tobacco and Cotton plants, the expression was distinguished in Cotton fiber rather than other plant cells [36]. *GhSCFP*, therefore, extensively used in transformation approaches for constitutive and global expression of fiber-specific genes. Moreover, possessing the specificity of the Tobacco seed coat, it can also be used for transformation studies of seed coat specific genes in dicot plants [37, 38].

On the other hand, *CpEXA1* expression under *CaMV35S*, being a developmental promoter [39] was not only found to be expressed equally in all plant tissues, but its expression was also relatively higher than that of the *GhSCFP* in leaves, sepals, and petals (Fig 9).

Another important fact to be noticed is that the relative expression of *FSEXPA1* gene increased gradually up to 18 DPA and then started to decrease in 24, 30 DPA old bolls, similarly, the expression pattern of *35SEXPA1* gene also increased and decreased in the same manner but the expression rate of former one was higher (Fig 8). This result can be correlated with the expression of the Expansin gene rather than promoter involvement. Since the Expansin gene specifically promotes cell wall elongation and it is evident that 5–18 DPA is the fiber elongation phase [15]. Likewise, the Expansin role is not reported to a significant extent during fiber initiation (0–5 DPA) and maturation/differentiation (20–45 DPA) phases [40].

## 5. Conclusion

Overexpression of Expansin gene, *CpEXPA1*, under *GhSCFP* and *CaMV35S* promoters, is found to be improving the fiber cellulose content when compared to that of the control group. *GhSCFP*, being a spatiotemporal promoter, expressed significantly in Cotton bolls specifically at 12,18 and 24 DPA. While *CaMV35S*, being a developmental promoter, continually expresses in all plant tissues (relative spatial expression) and during 12,18,24 and 30 DPA (temporally). It has now hypothesized that *GhSCFP* could contribute more efficiently to fiber-related gene expression than *CaMV35S*.

## Supporting information

S1 File (ZIP)
