# Supplementary figures and images for: Comparative analysis of Constitutive and fiber-specific promoters under the expression pattern of Expansin gene in transgenic Cotton
Source: PLoS One. 2020 Mar 18;15(3):e0230519. doi: 10.1371/journal.pone.0230519 (PMC7080281; doi:10.1371/journal.pone.0230519)

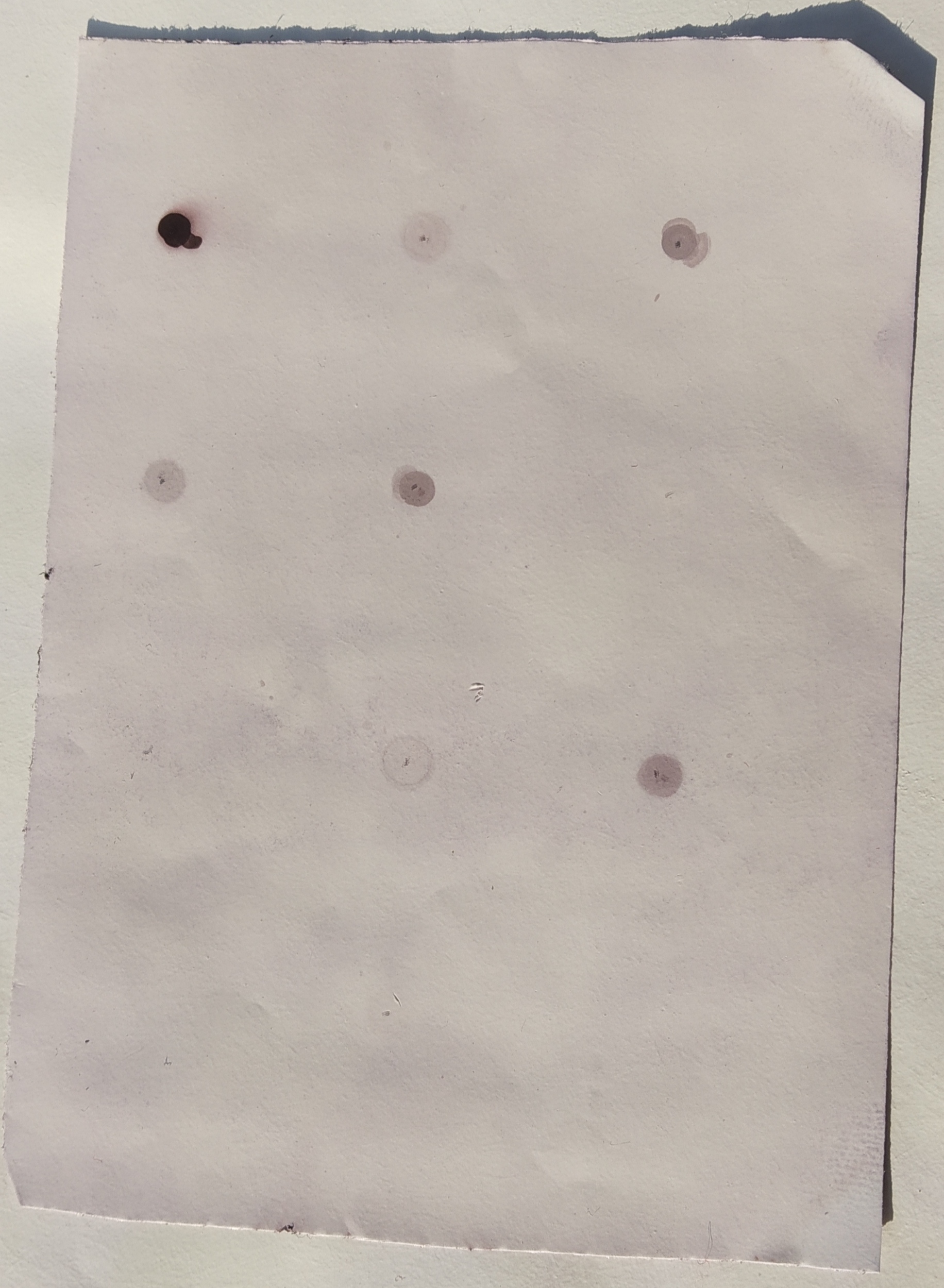

Supplement: S1 File — (ZIP) [file pone.0230519.s001.zip › IMG_20200103_090138.jpg]

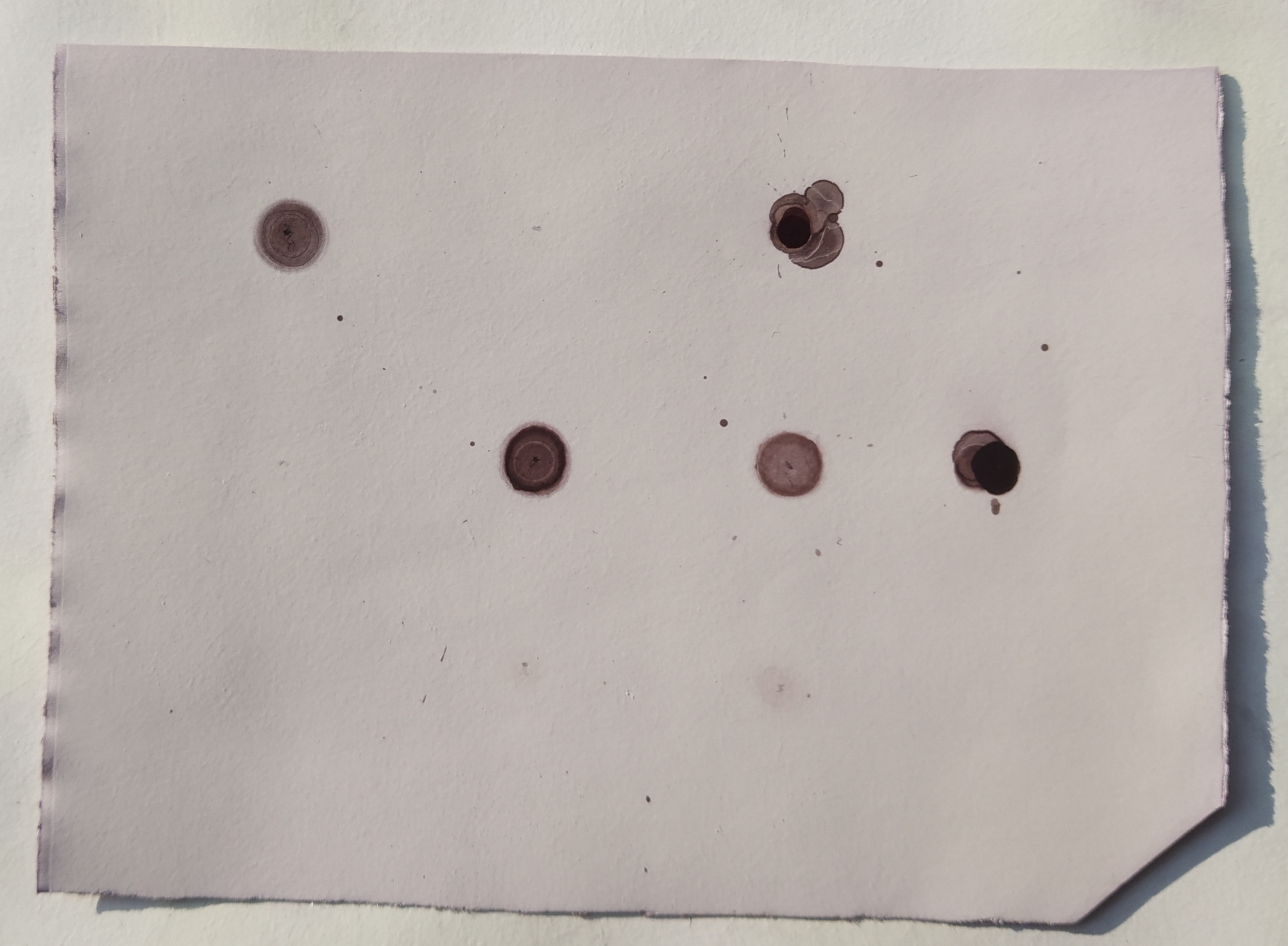

Supplement: S1 File — (ZIP) [file pone.0230519.s001.zip › IMG_20200103_090251.jpg]

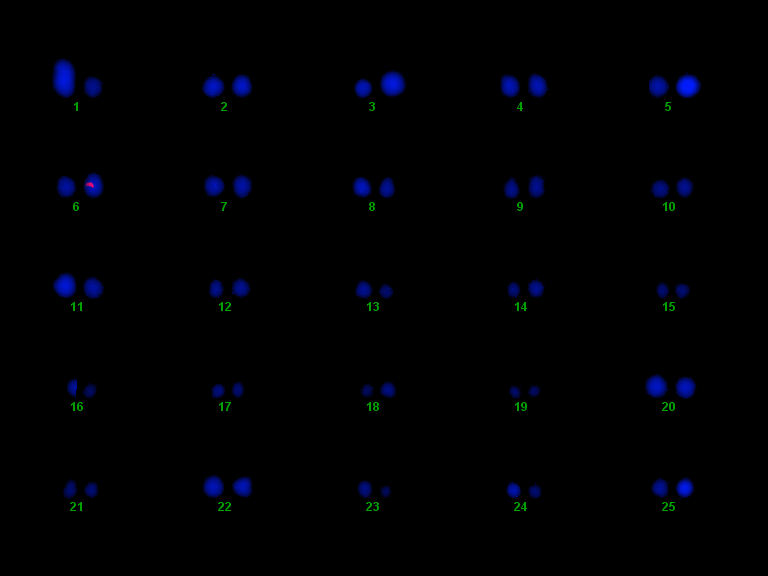

Supplement: S1 File — (ZIP) [file pone.0230519.s001.zip › c6.tif]

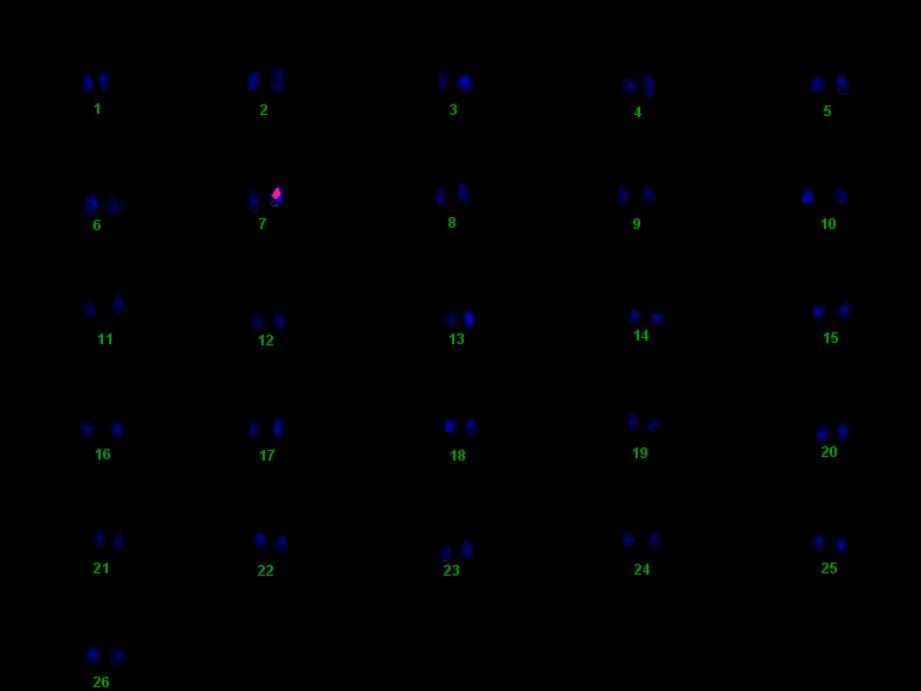

Supplement: S1 File — (ZIP) [file pone.0230519.s001.zip › c7.jpg]

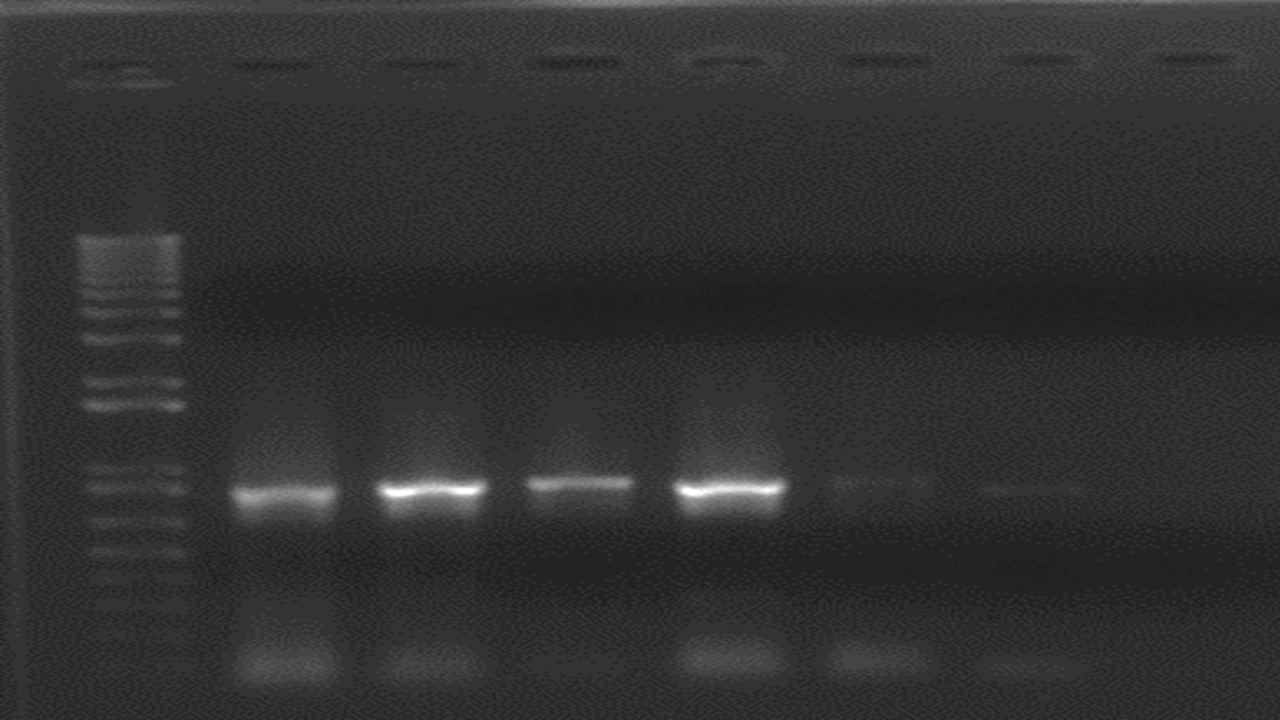

Supplement: S1 File — (ZIP) [file pone.0230519.s001.zip › gel (35S).tif]

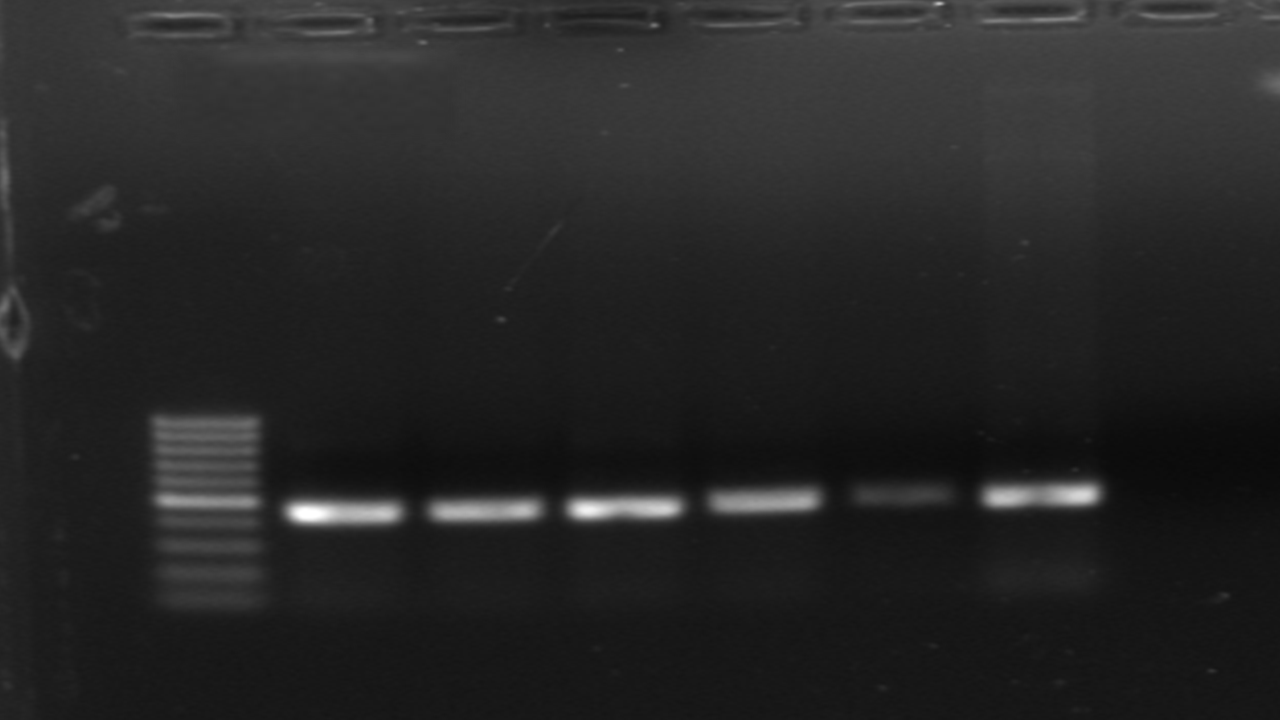

Supplement: S1 File — (ZIP) [file pone.0230519.s001.zip › gel (FS).tif]

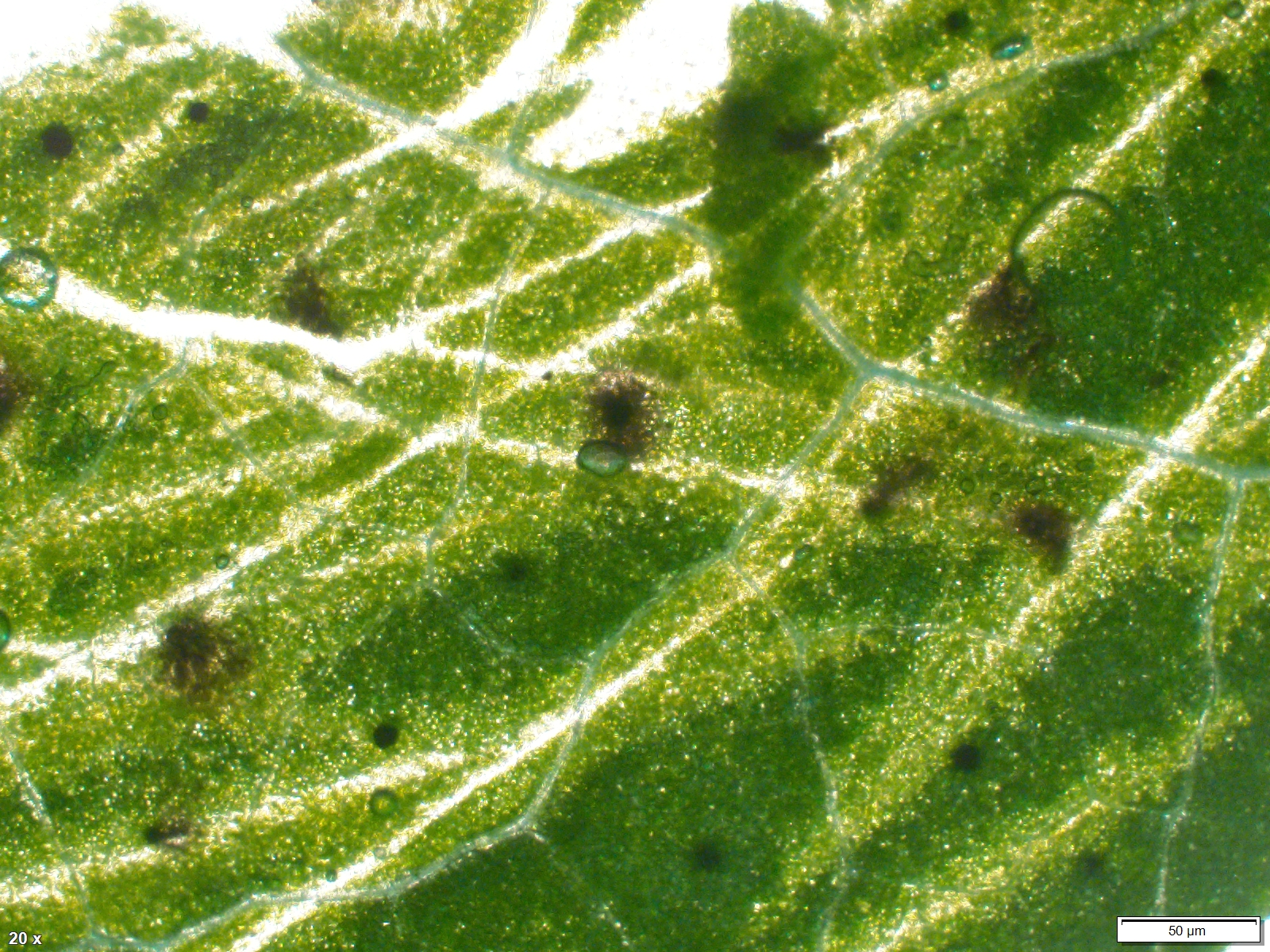

Supplement: S1 File — (ZIP) [file pone.0230519.s001.zip › Image_1917.jpg]

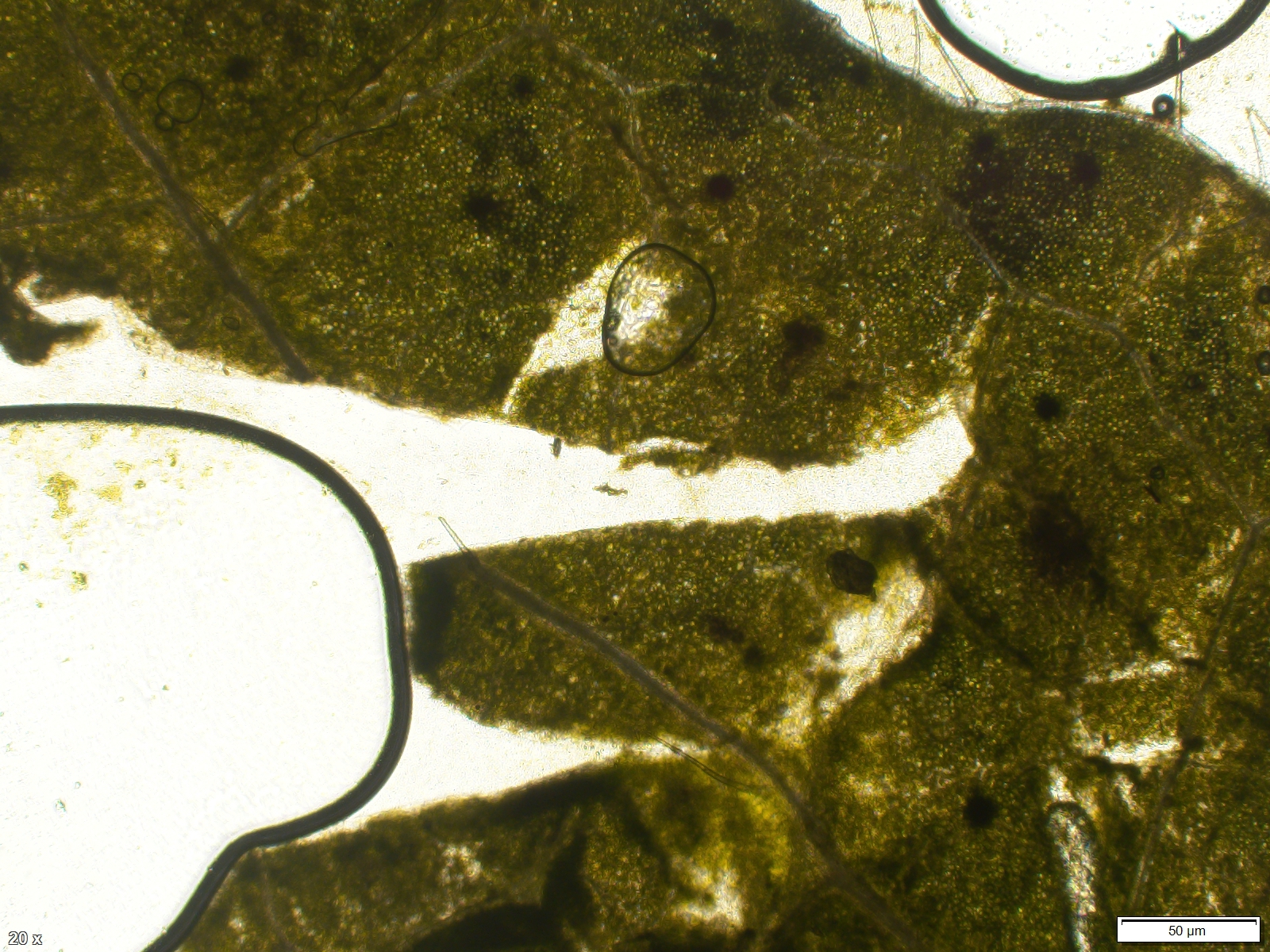

Supplement: S1 File — (ZIP) [file pone.0230519.s001.zip › Image_1918.jpg]

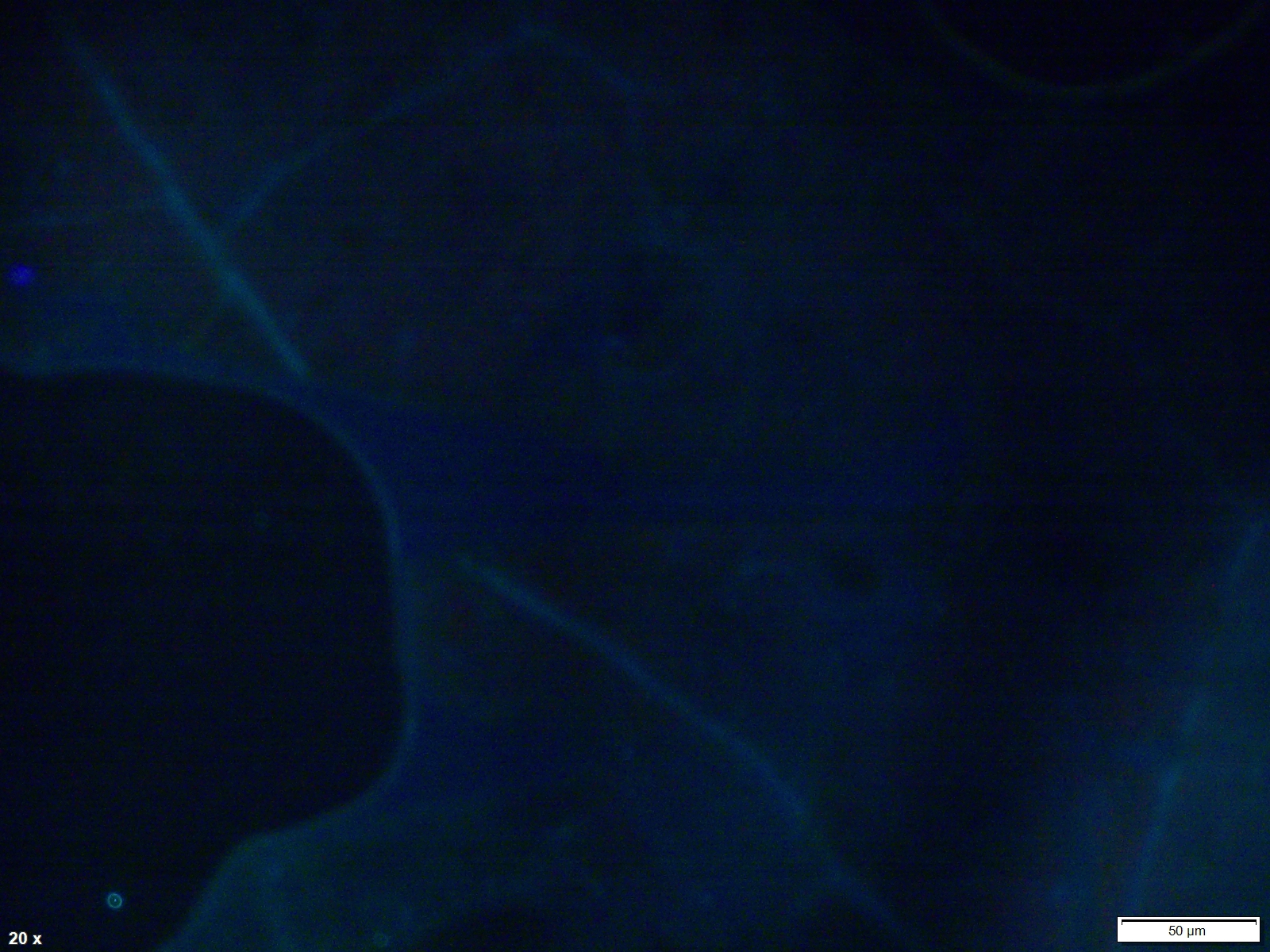

Supplement: S1 File — (ZIP) [file pone.0230519.s001.zip › Image_1919.jpg]

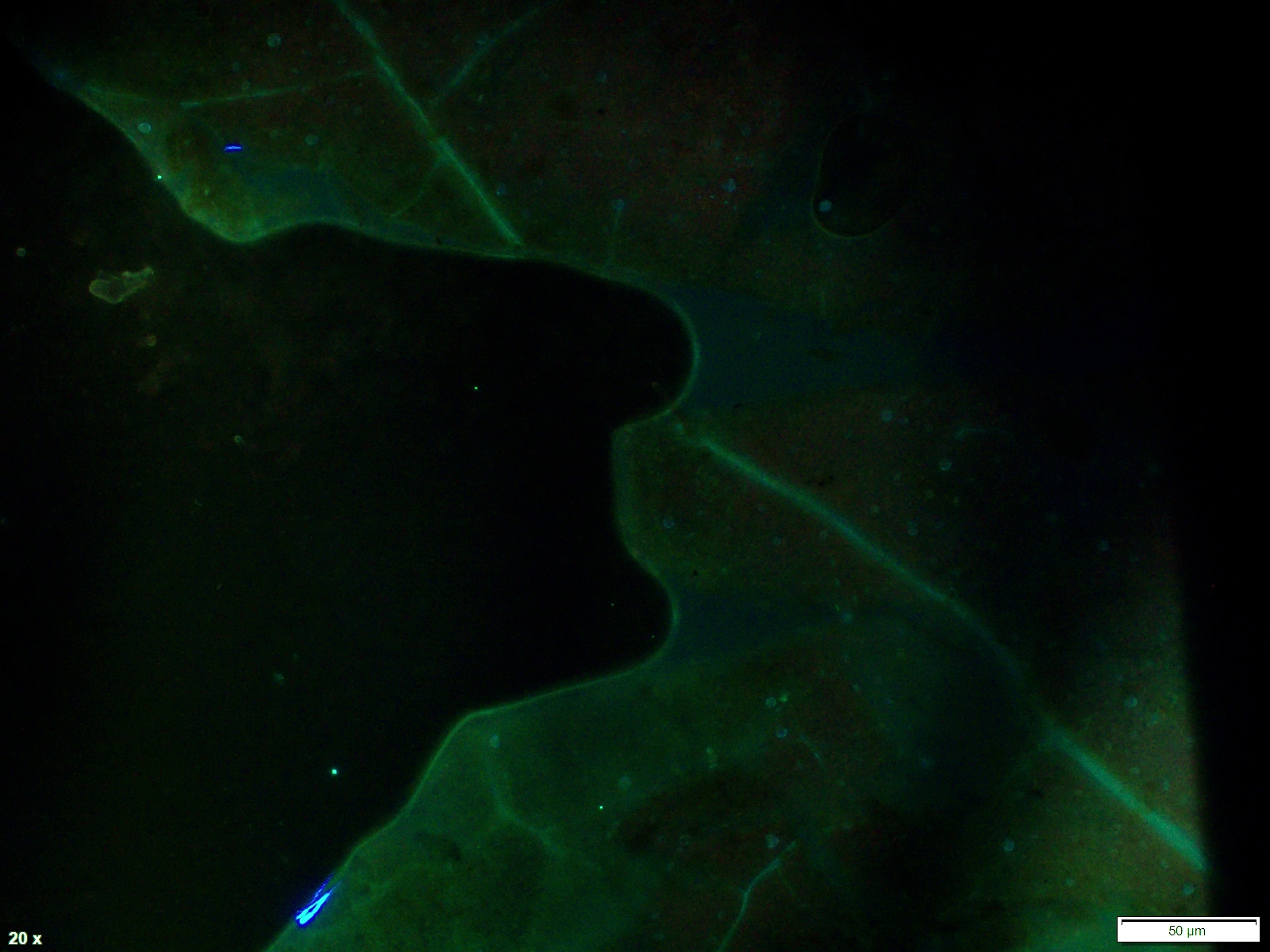

Supplement: S1 File — (ZIP) [file pone.0230519.s001.zip › Image_1923.jpg]

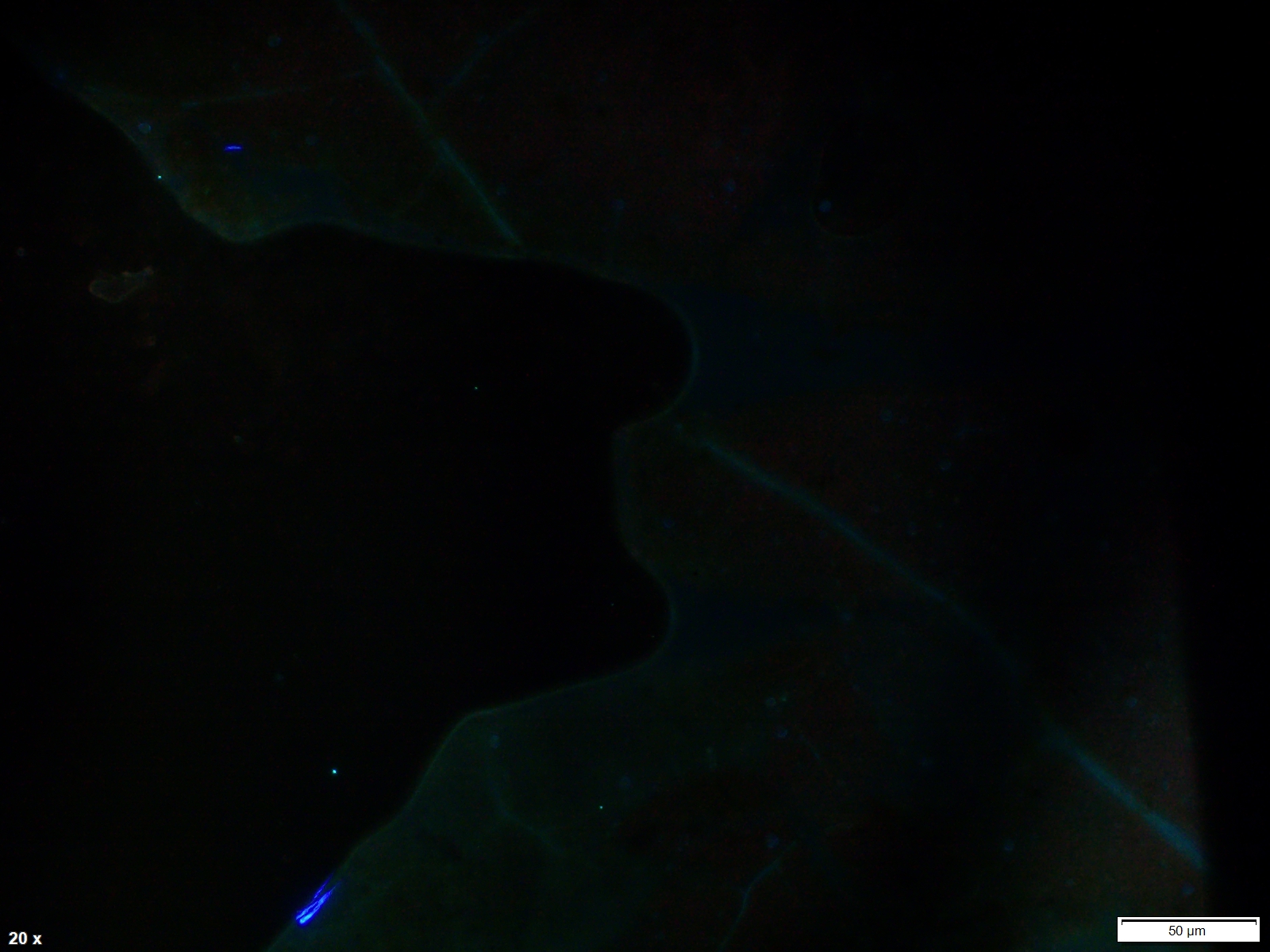

Supplement: S1 File — (ZIP) [file pone.0230519.s001.zip › Image_1924.jpg]

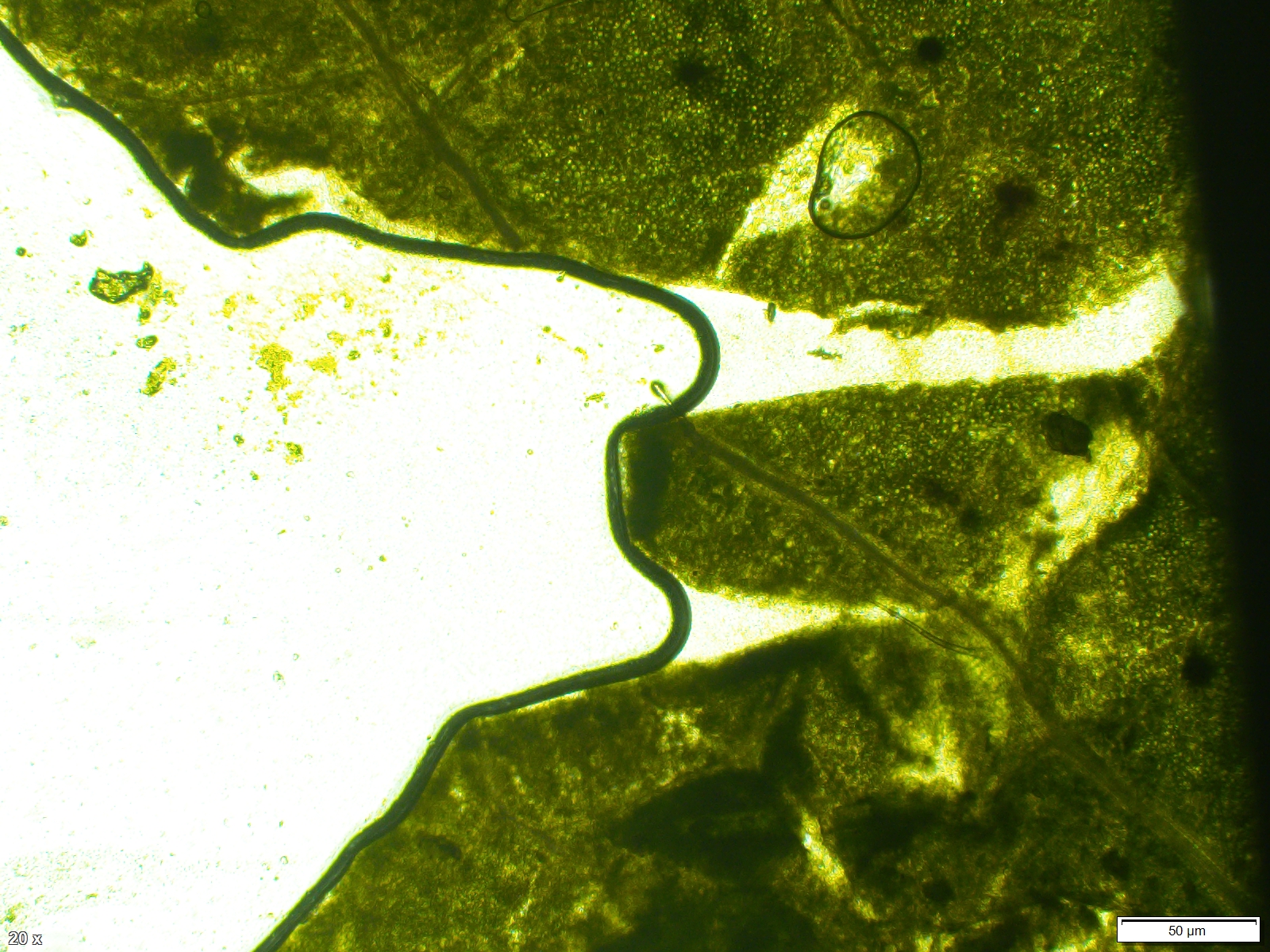

Supplement: S1 File — (ZIP) [file pone.0230519.s001.zip › Image_1925.jpg]

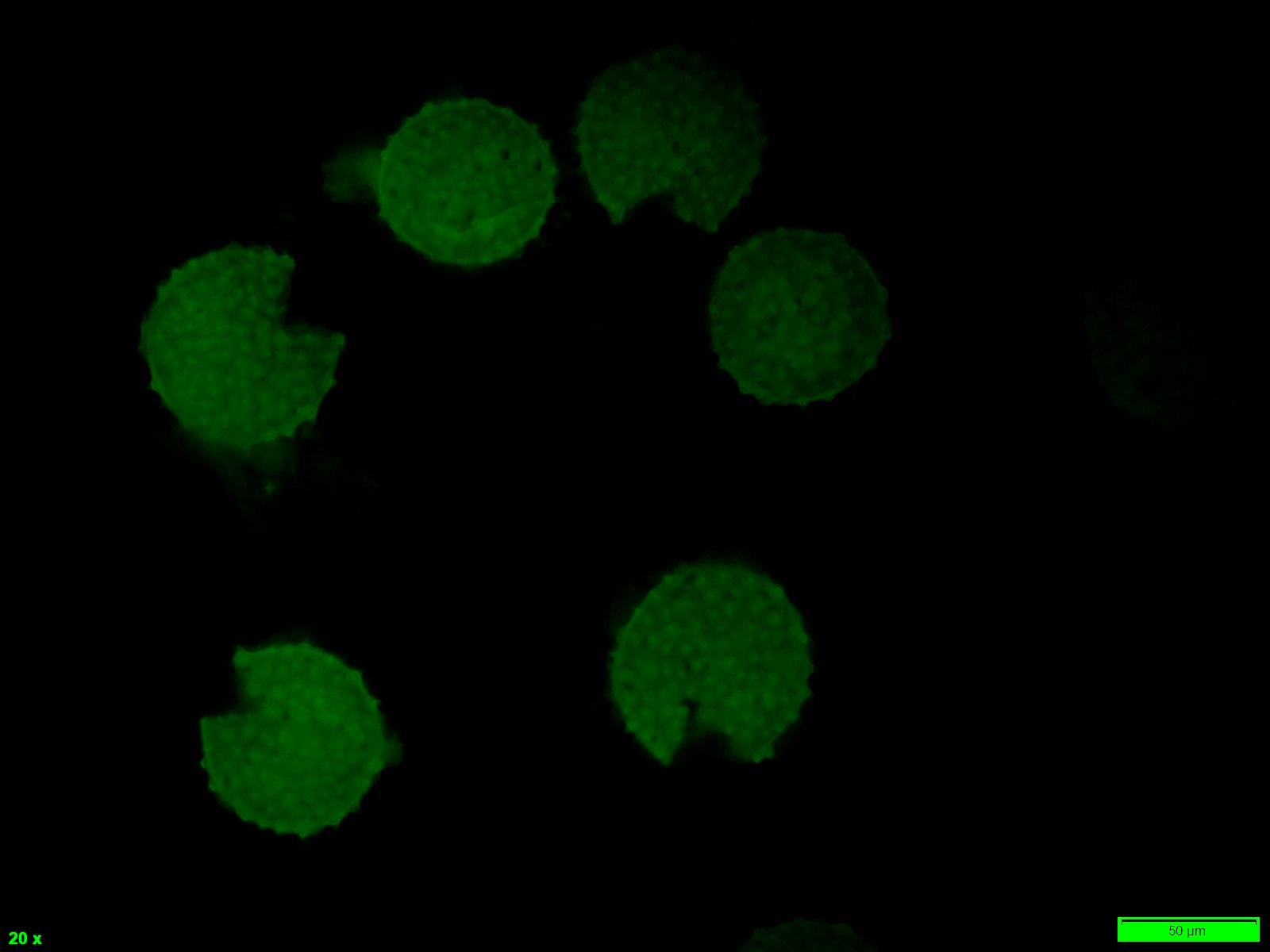

Supplement: S1 File — (ZIP) [file pone.0230519.s001.zip › Image_1938.jpg]

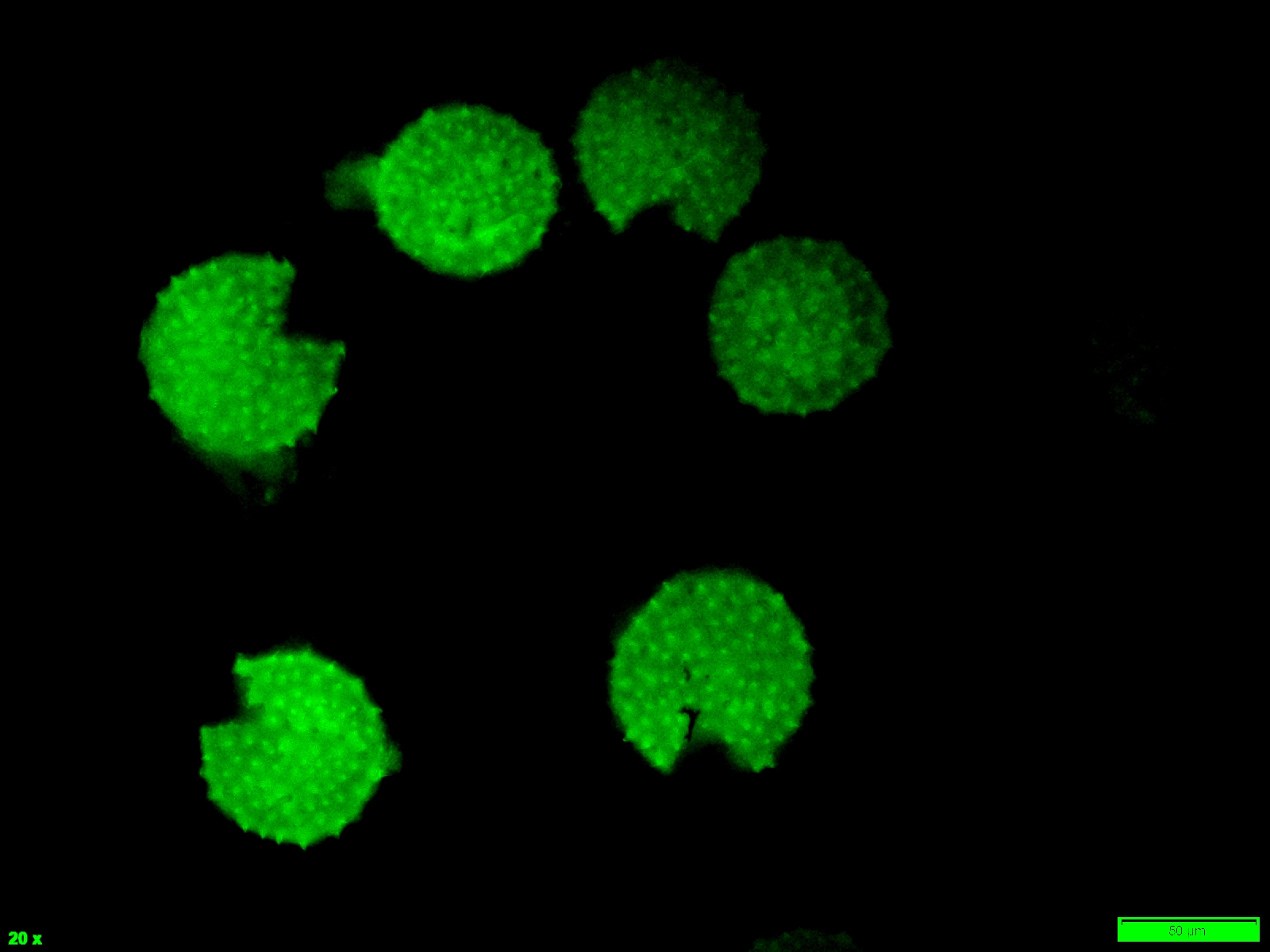

Supplement: S1 File — (ZIP) [file pone.0230519.s001.zip › Image_1939.jpg]

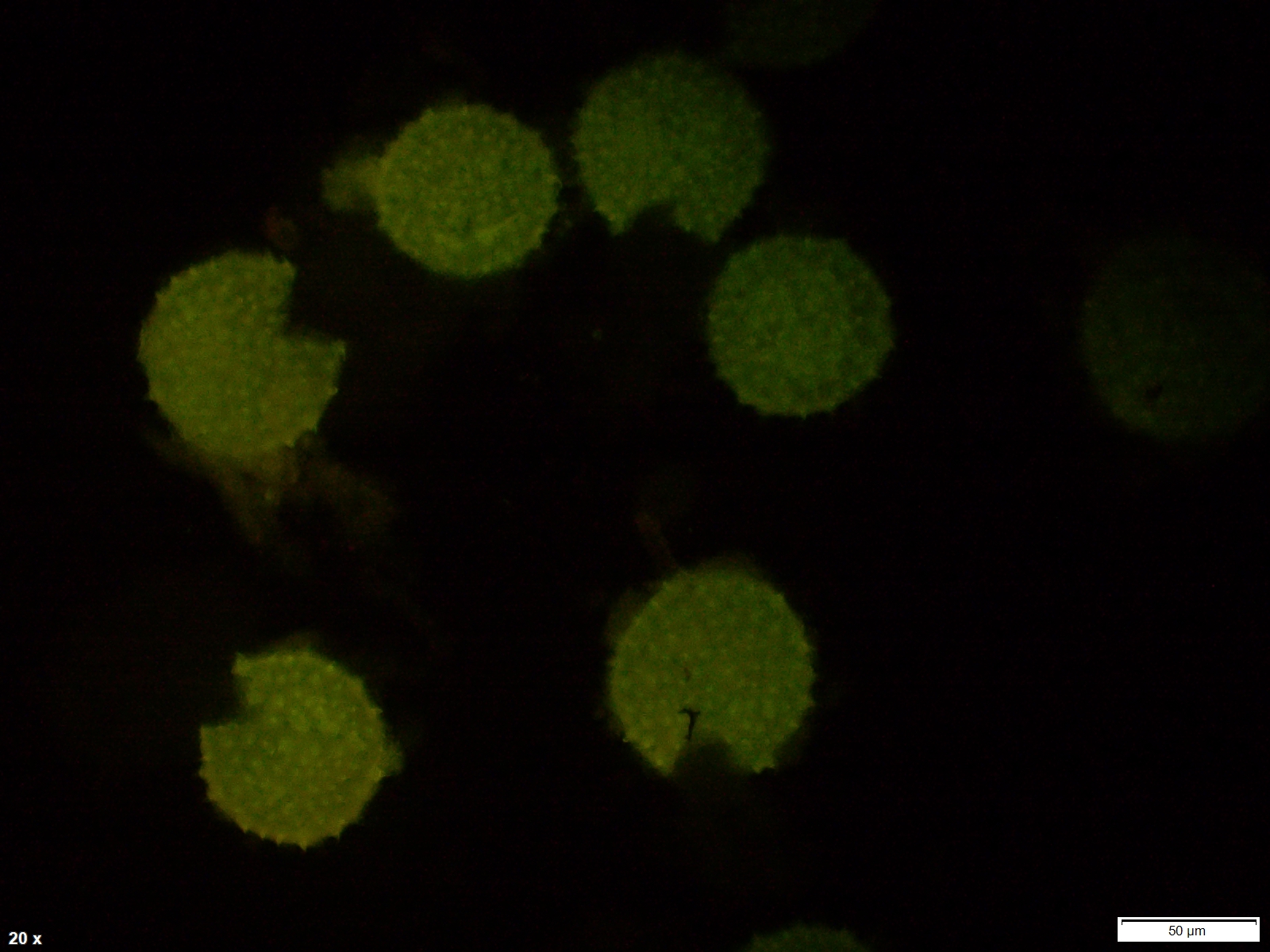

Supplement: S1 File — (ZIP) [file pone.0230519.s001.zip › Image_1940.jpg]

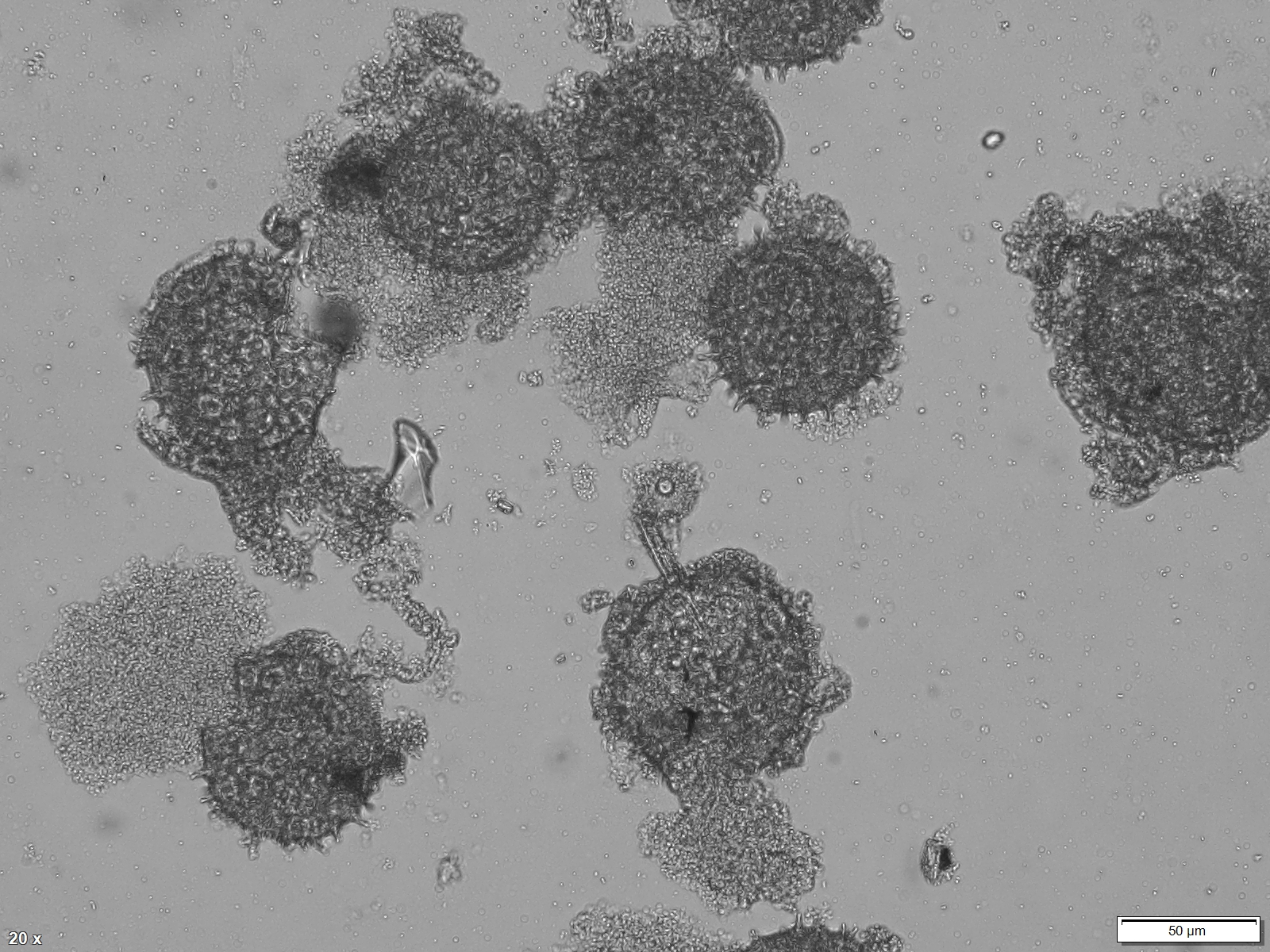

Supplement: S1 File — (ZIP) [file pone.0230519.s001.zip › Image_1941.jpg]

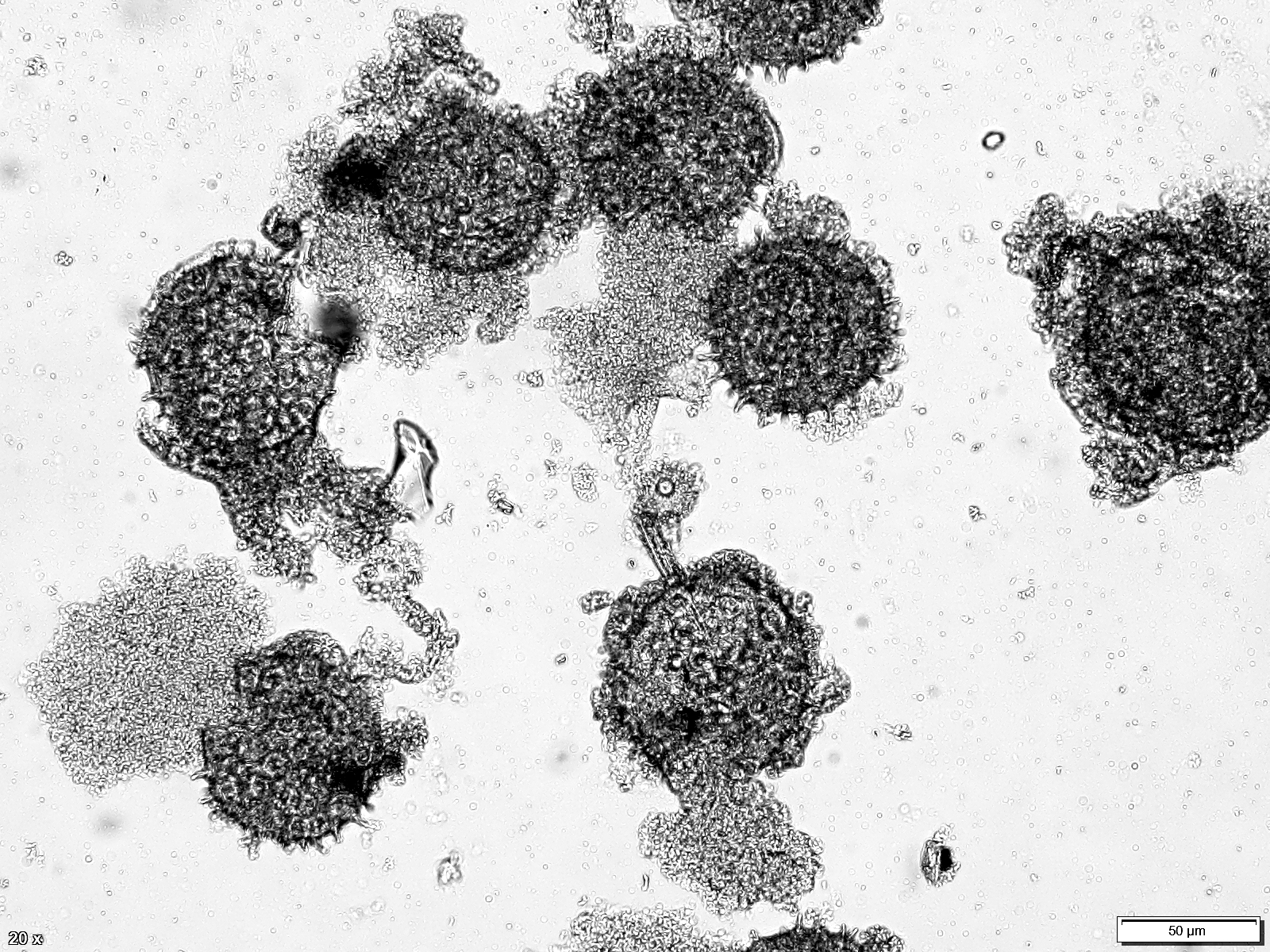

Supplement: S1 File — (ZIP) [file pone.0230519.s001.zip › Image_1943.jpg]

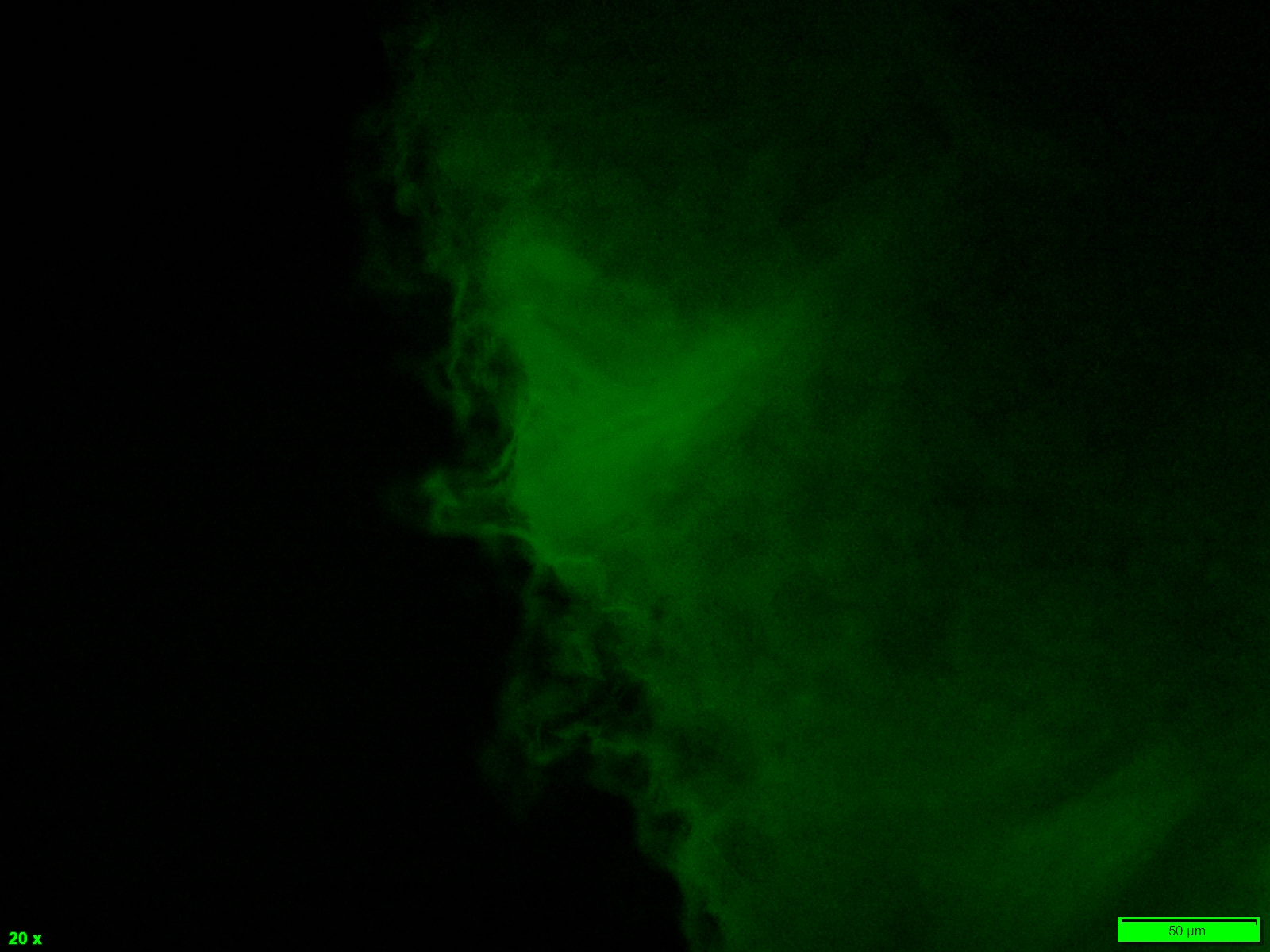

Supplement: S1 File — (ZIP) [file pone.0230519.s001.zip › Image_1952.jpg]

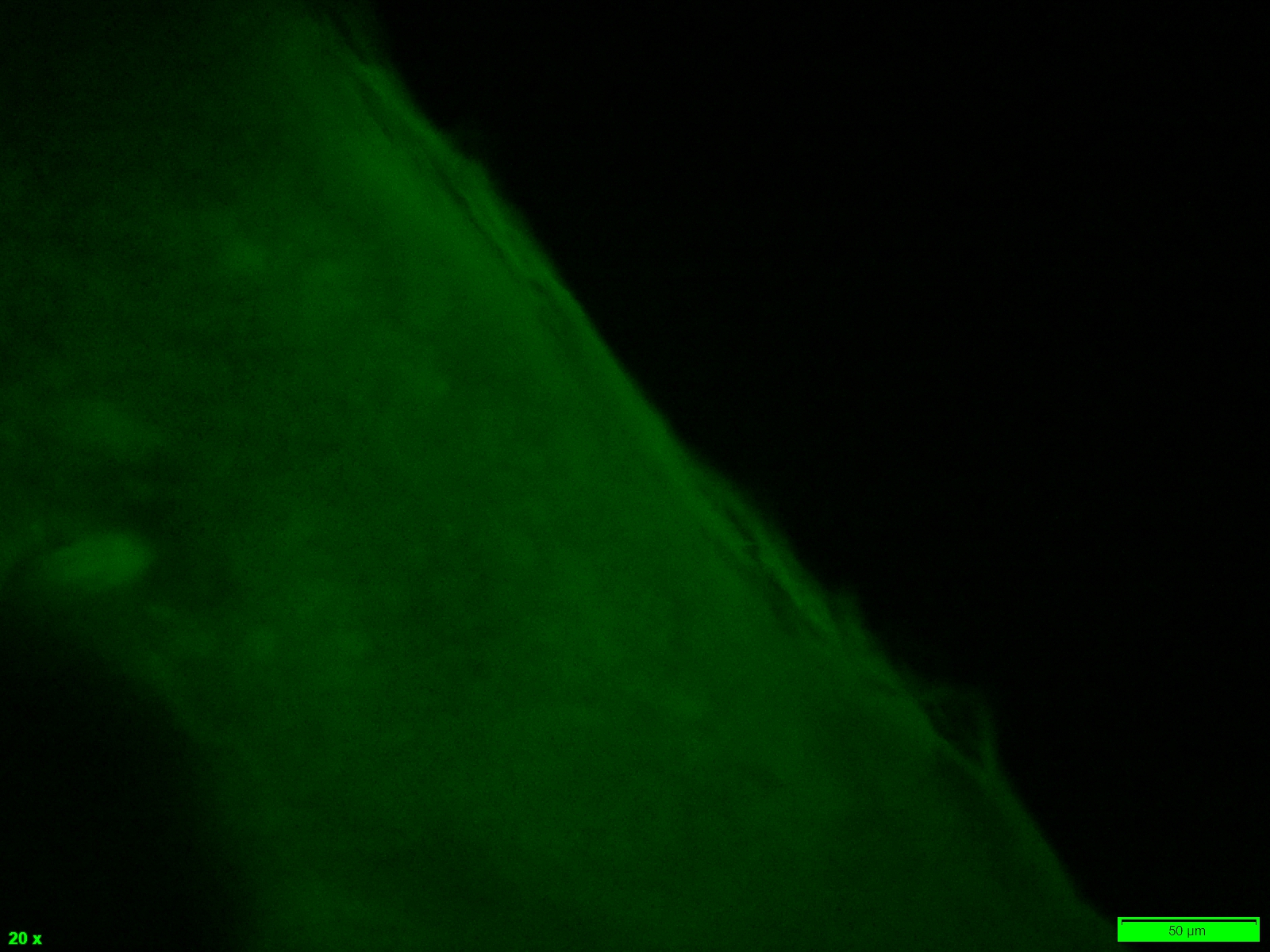

Supplement: S1 File — (ZIP) [file pone.0230519.s001.zip › Image_1955.jpg]

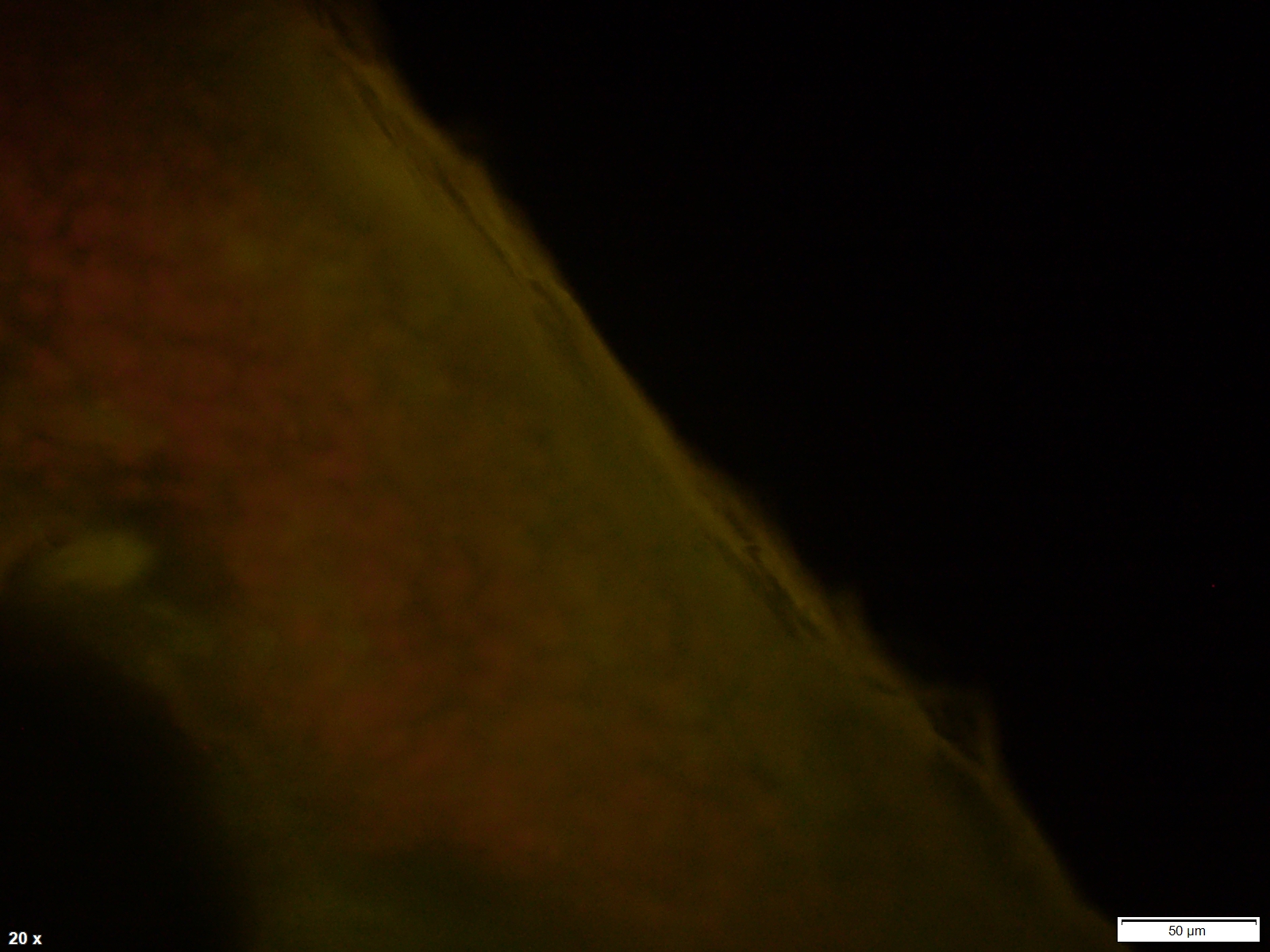

Supplement: S1 File — (ZIP) [file pone.0230519.s001.zip › Image_1956.jpg]

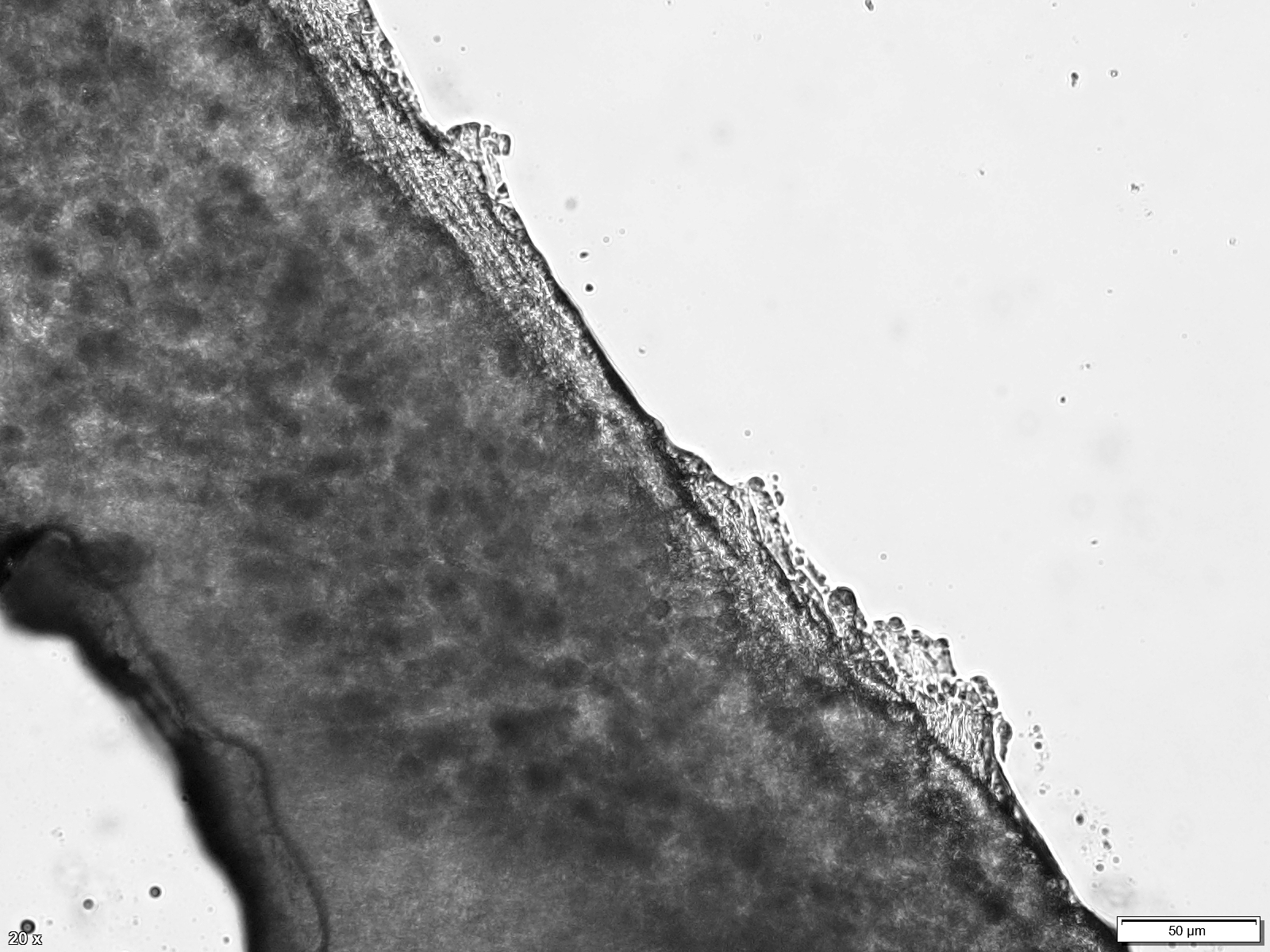

Supplement: S1 File — (ZIP) [file pone.0230519.s001.zip › Image_1957.jpg]

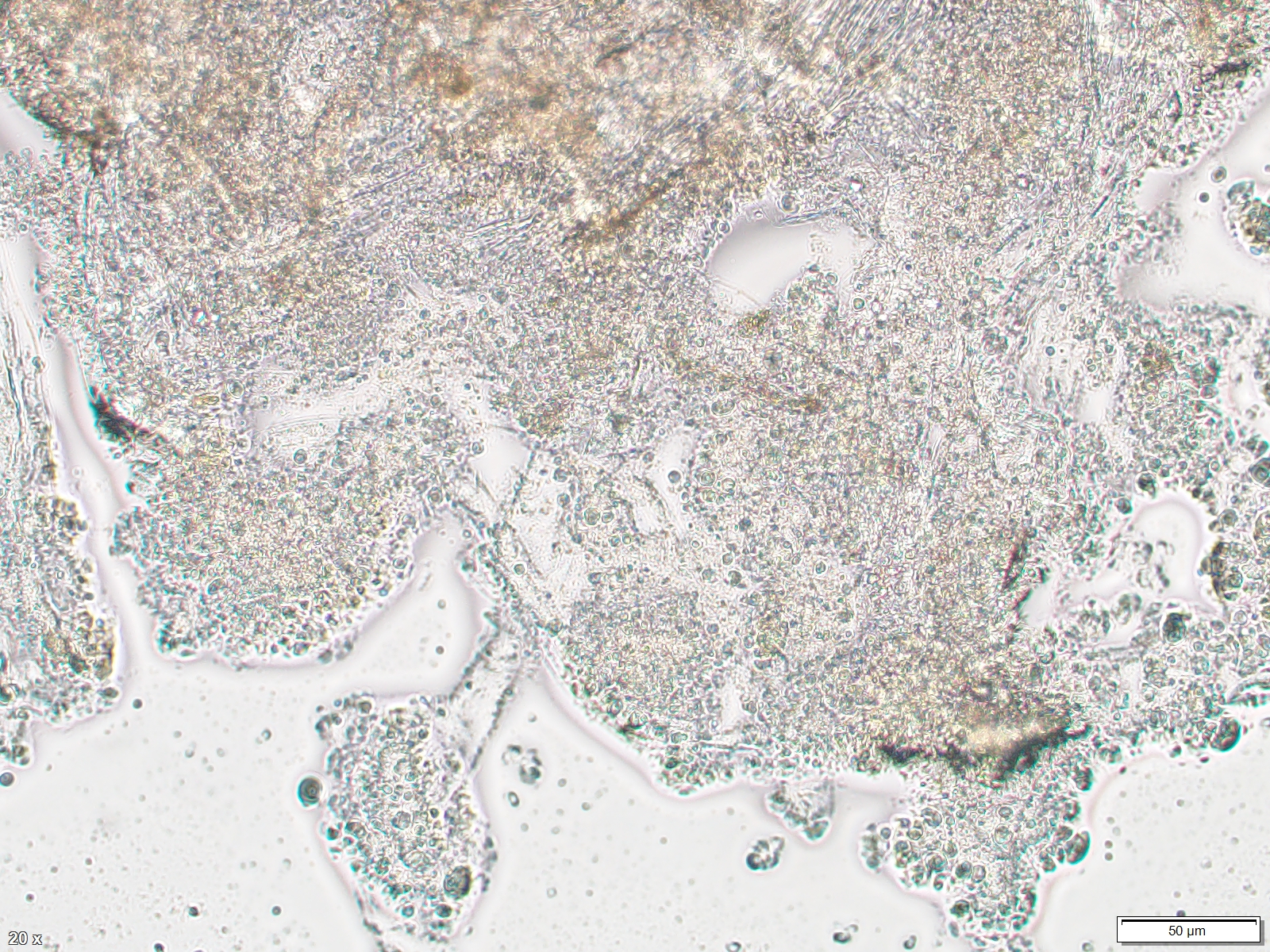

Supplement: S1 File — (ZIP) [file pone.0230519.s001.zip › Image_1959.jpg]

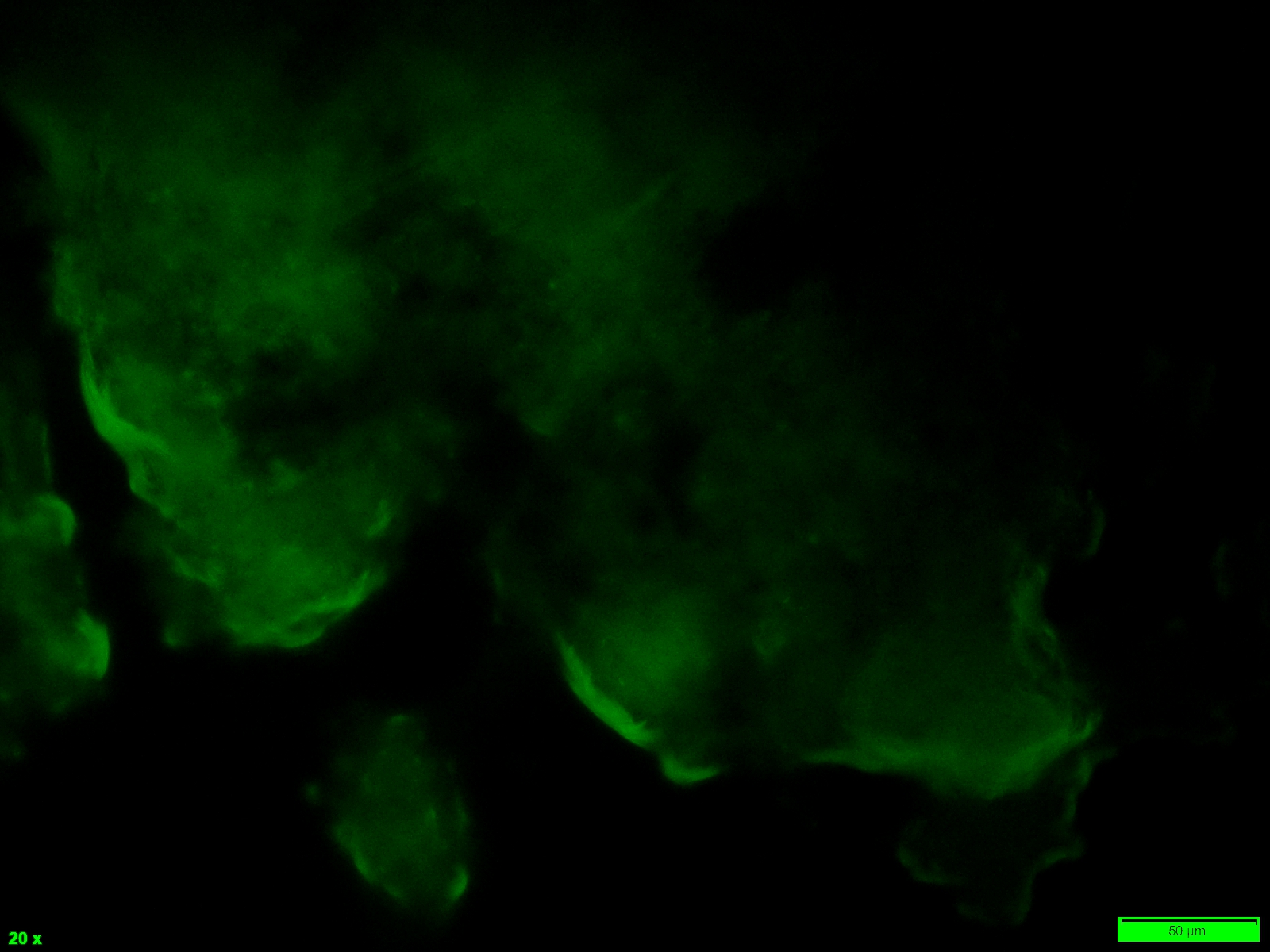

Supplement: S1 File — (ZIP) [file pone.0230519.s001.zip › Image_1960.jpg]

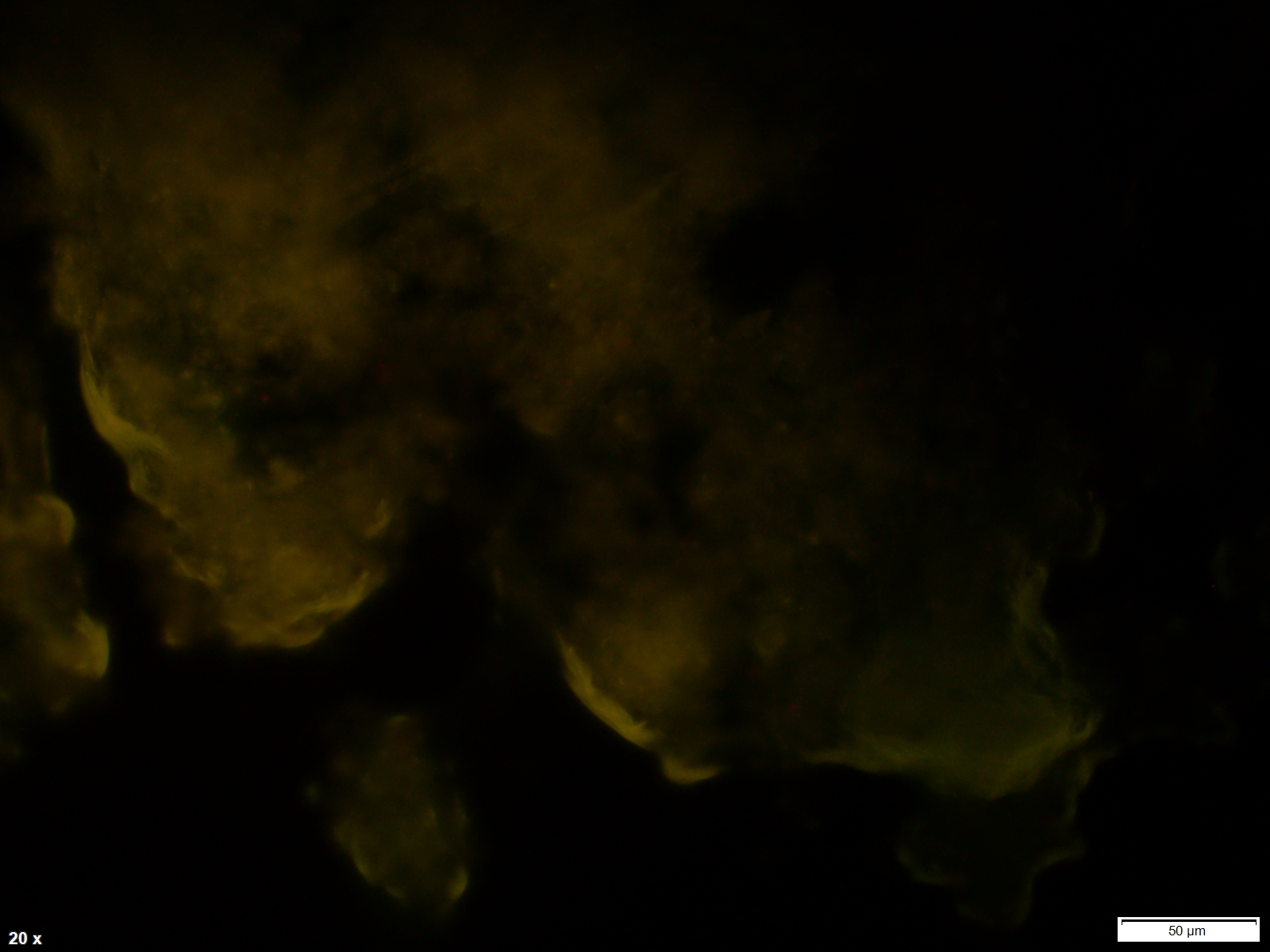

Supplement: S1 File — (ZIP) [file pone.0230519.s001.zip › Image_1961.jpg]

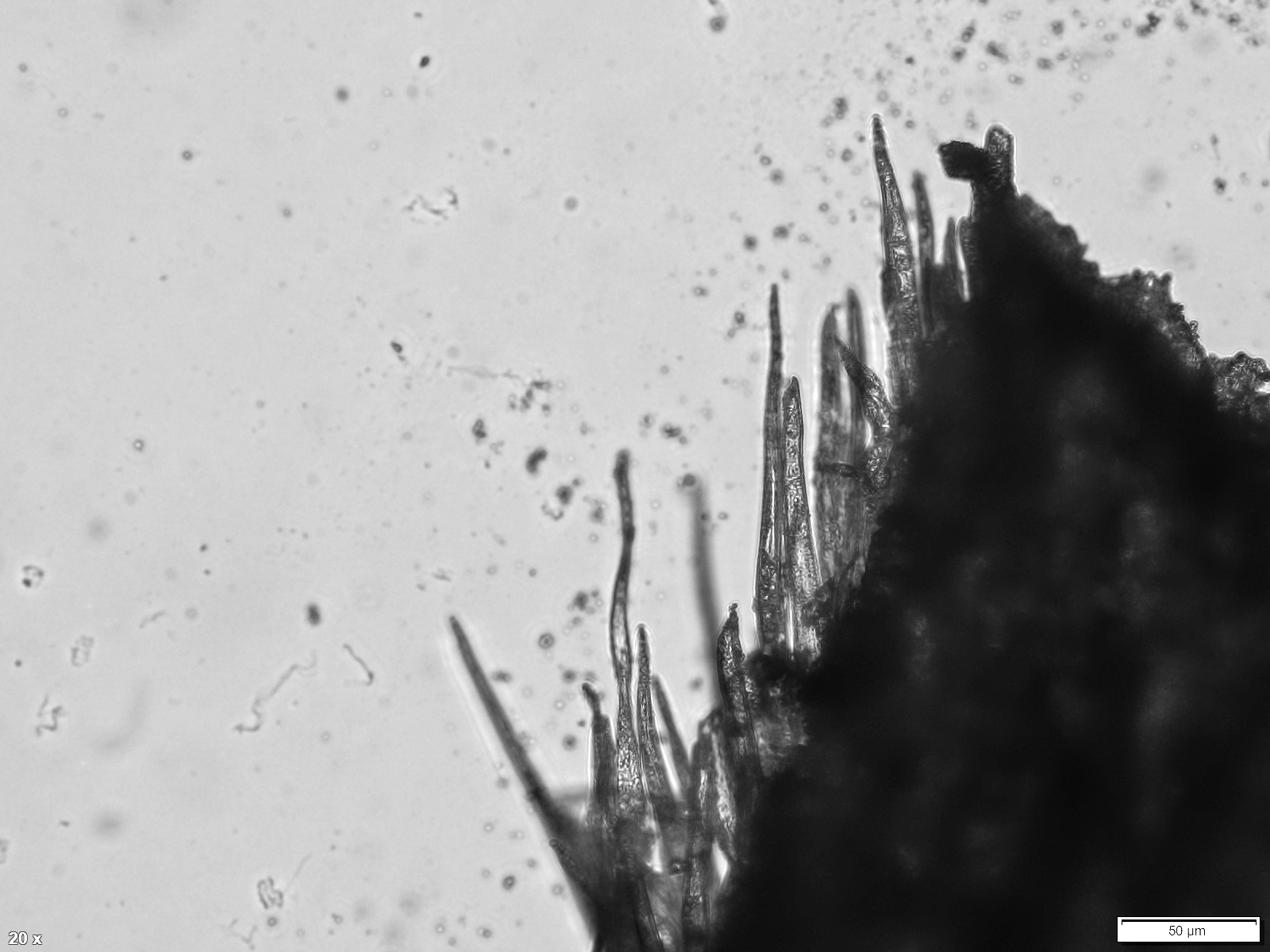

Supplement: S1 File — (ZIP) [file pone.0230519.s001.zip › Image_1967.jpg]

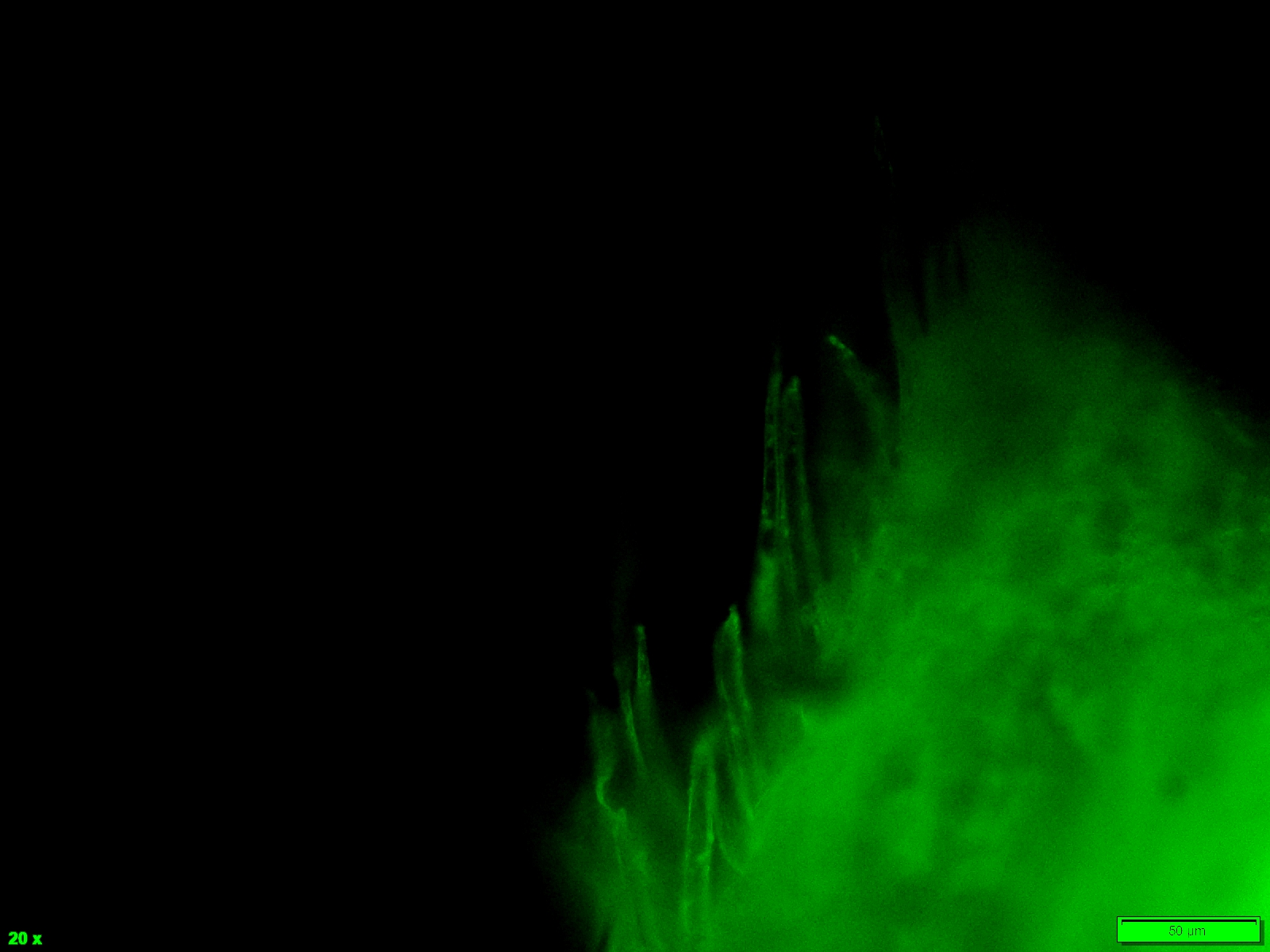

Supplement: S1 File — (ZIP) [file pone.0230519.s001.zip › Image_1969 (1).jpg]

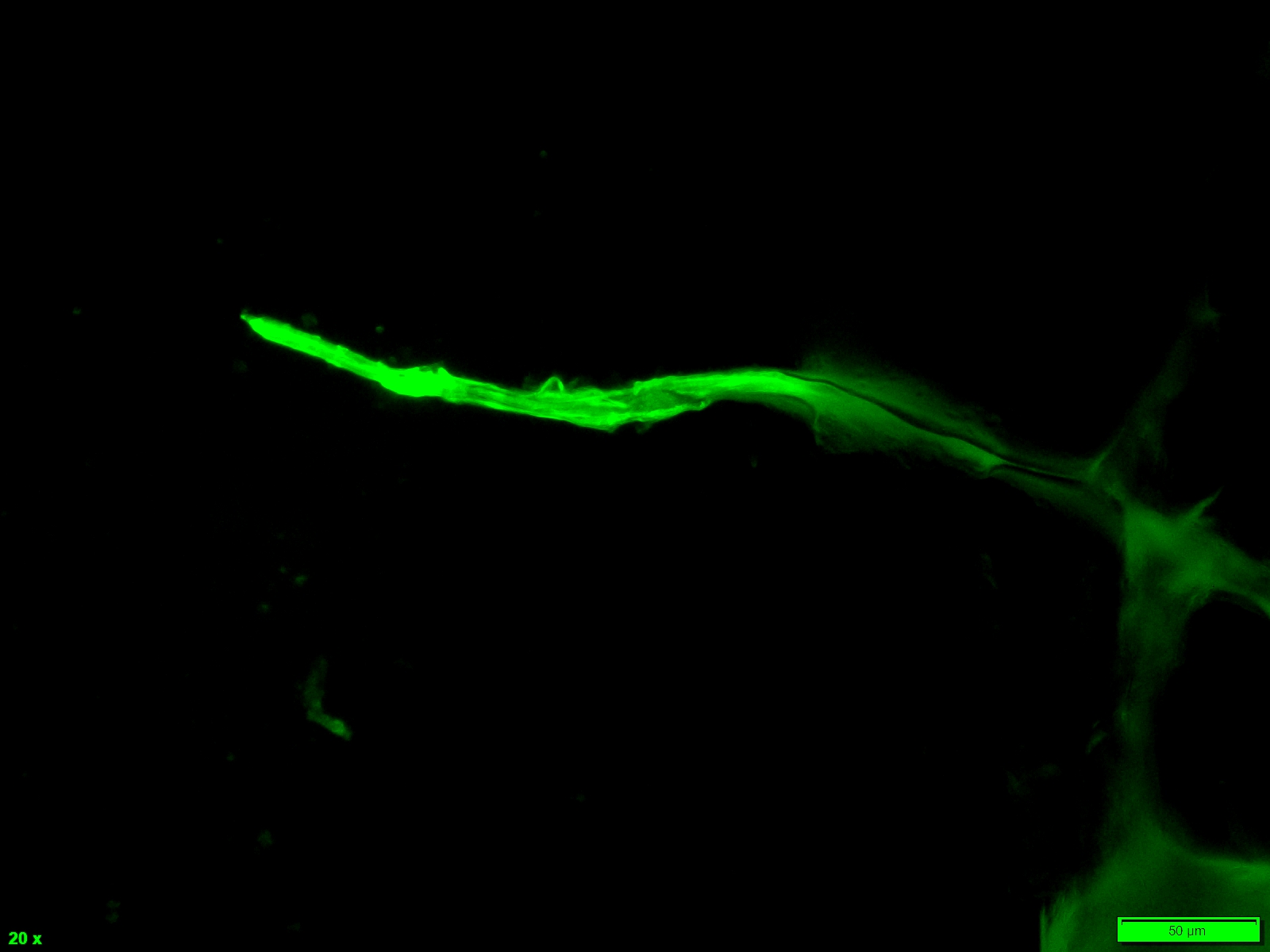

Supplement: S1 File — (ZIP) [file pone.0230519.s001.zip › Image_1982.jpg]

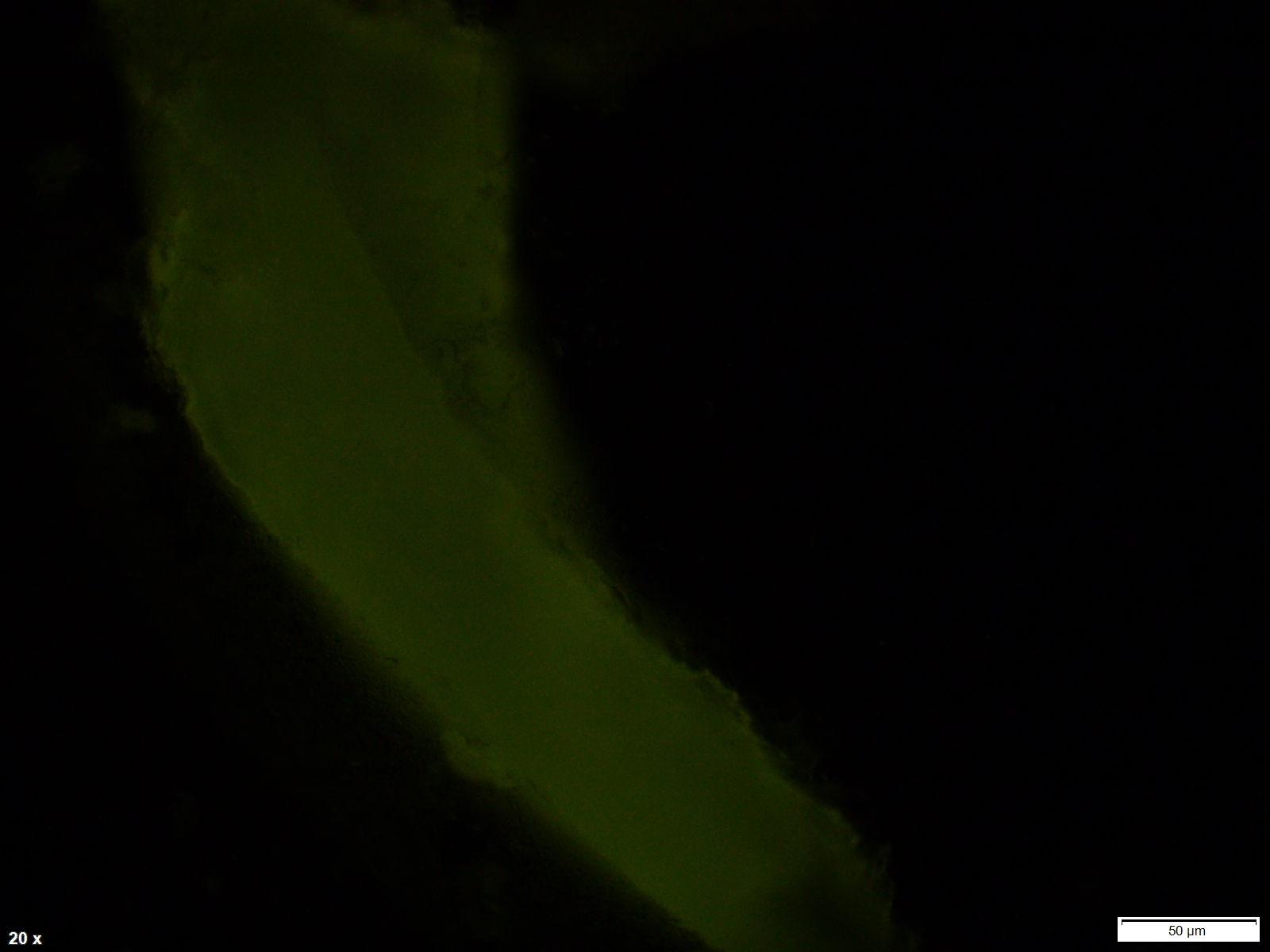

Supplement: S1 File — (ZIP) [file pone.0230519.s001.zip › Image_1995.jpg]

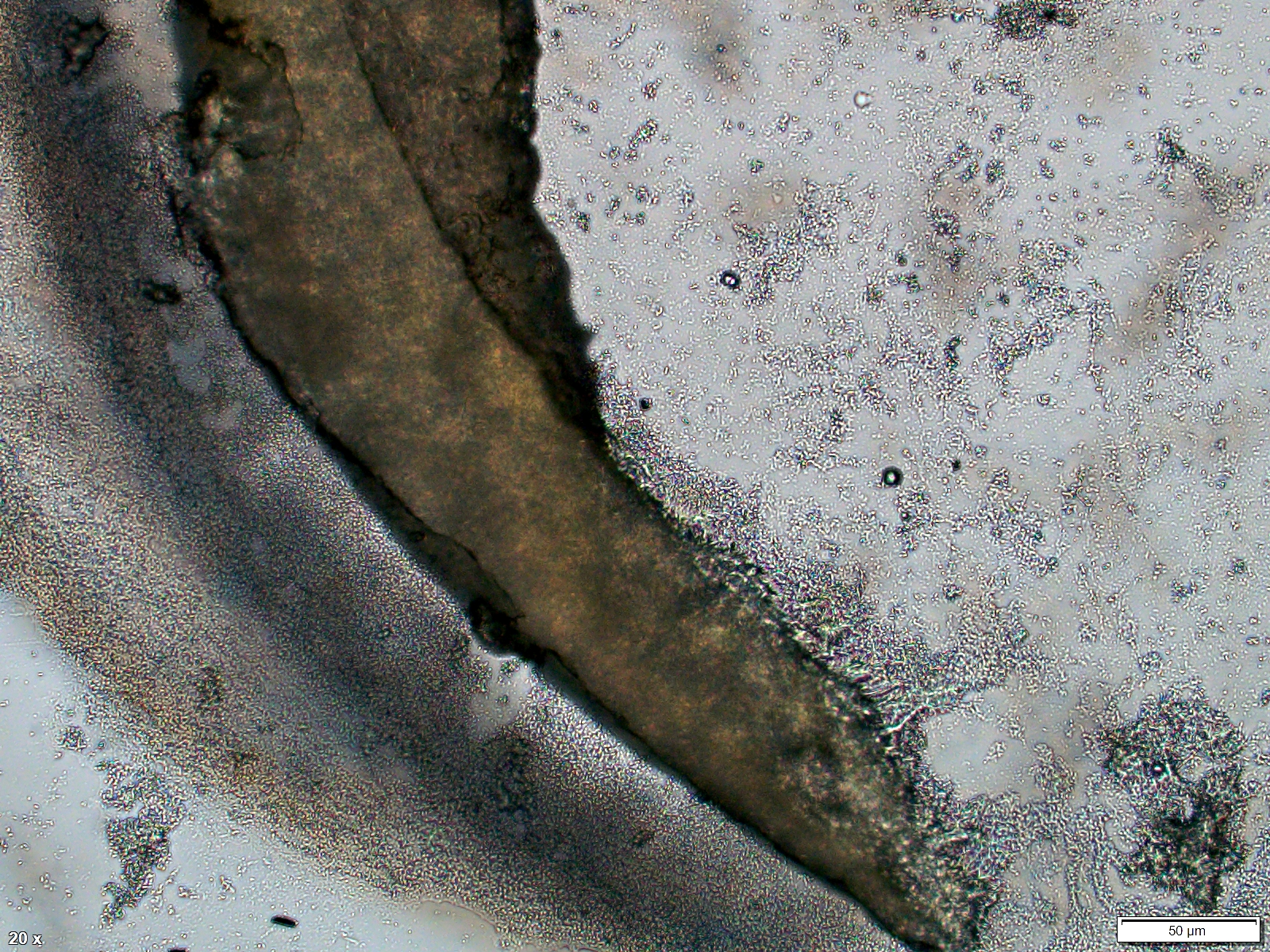

Supplement: S1 File — (ZIP) [file pone.0230519.s001.zip › Image_1997.jpg]

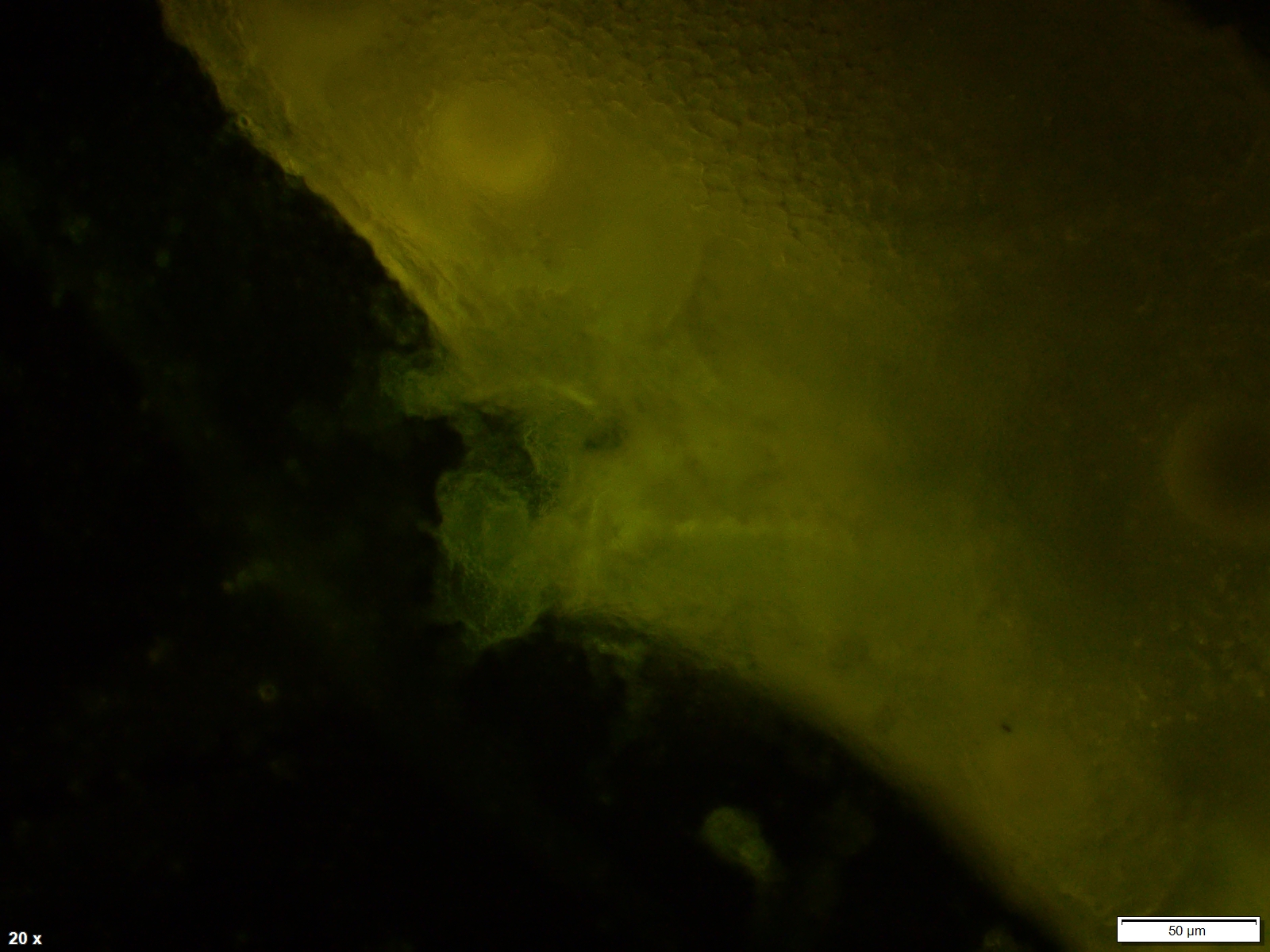

Supplement: S1 File — (ZIP) [file pone.0230519.s001.zip › Image_2004.jpg]

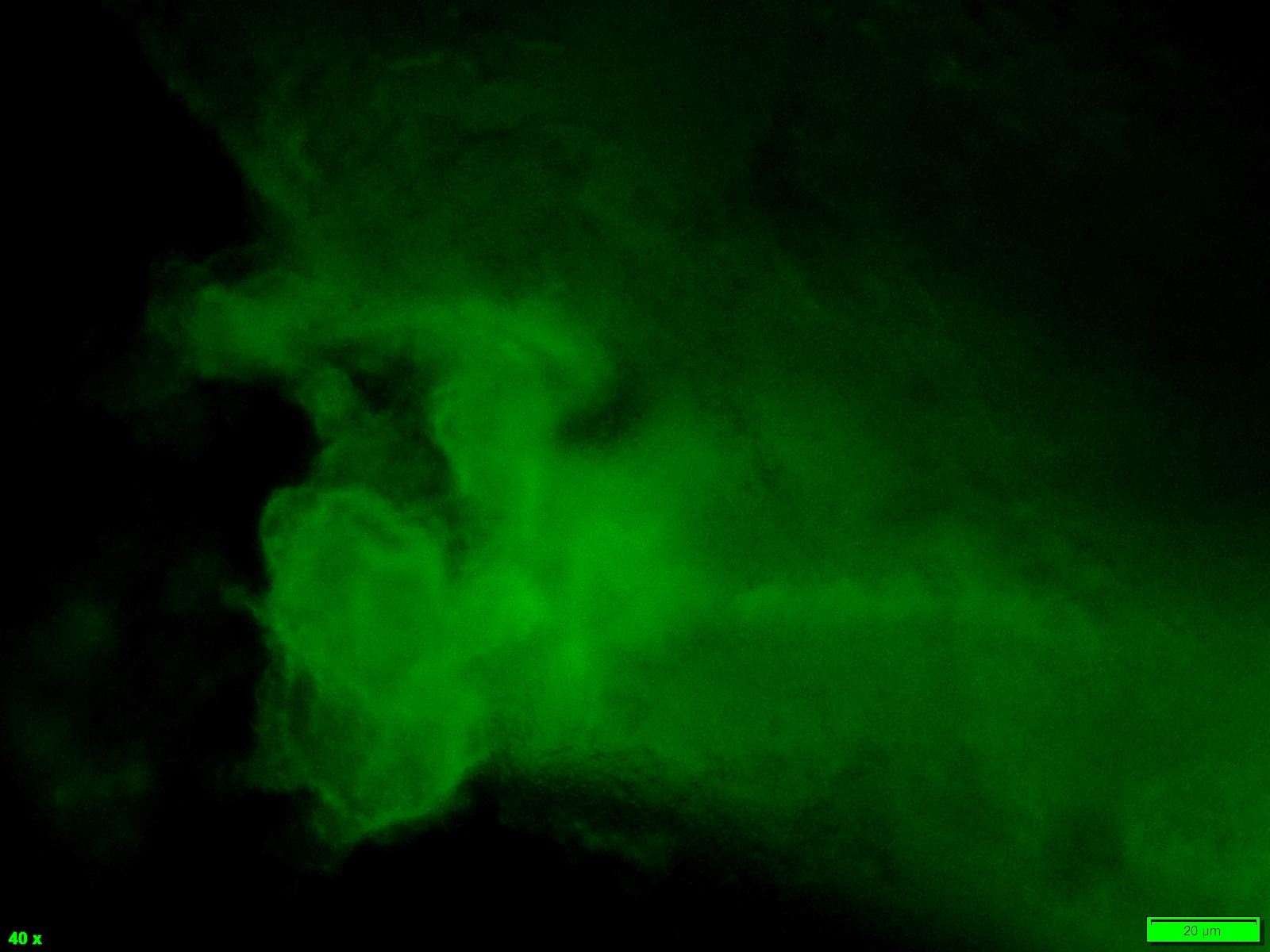

Supplement: S1 File — (ZIP) [file pone.0230519.s001.zip › Image_2005.jpg]

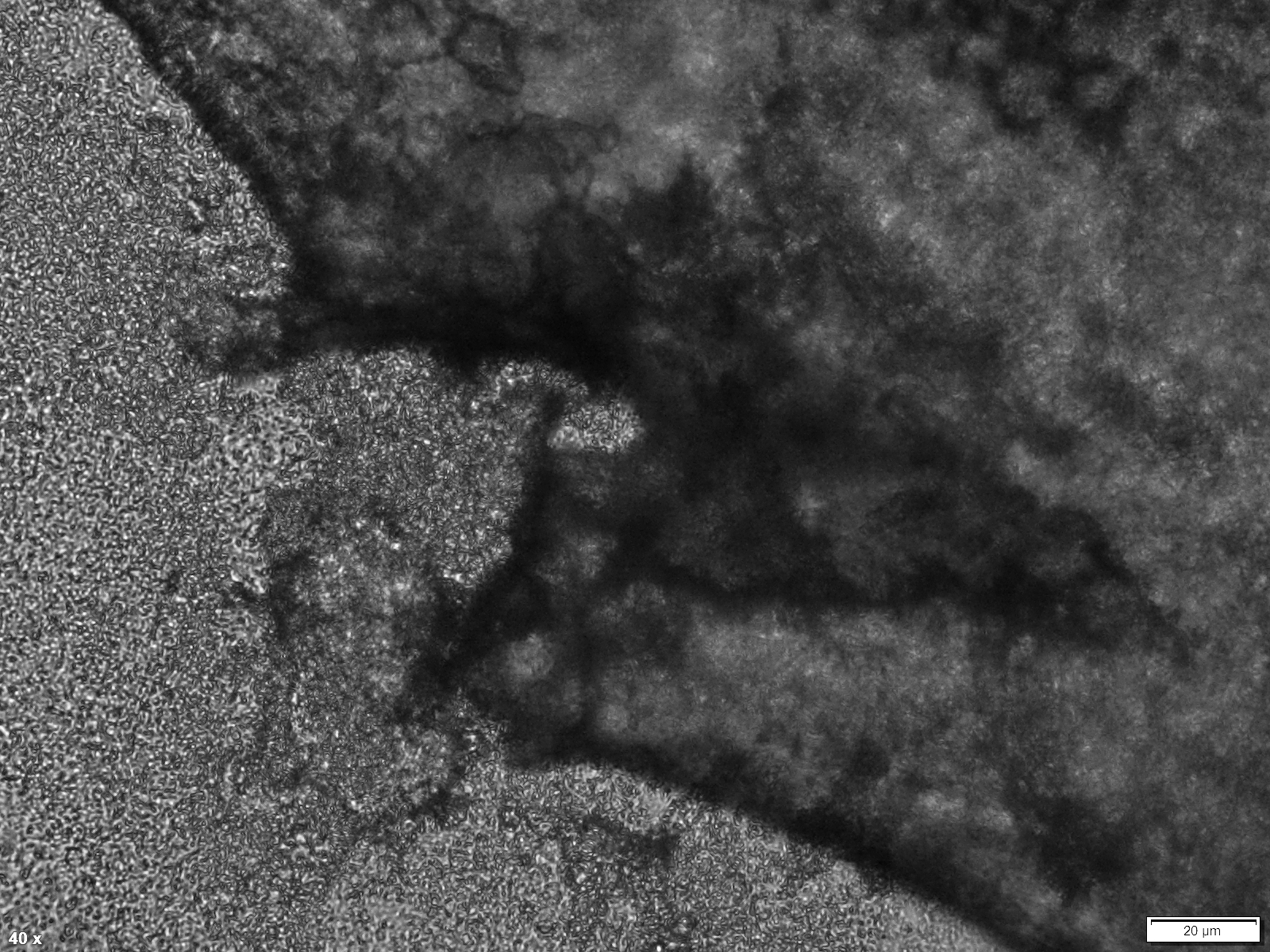

Supplement: S1 File — (ZIP) [file pone.0230519.s001.zip › Image_2006.jpg]

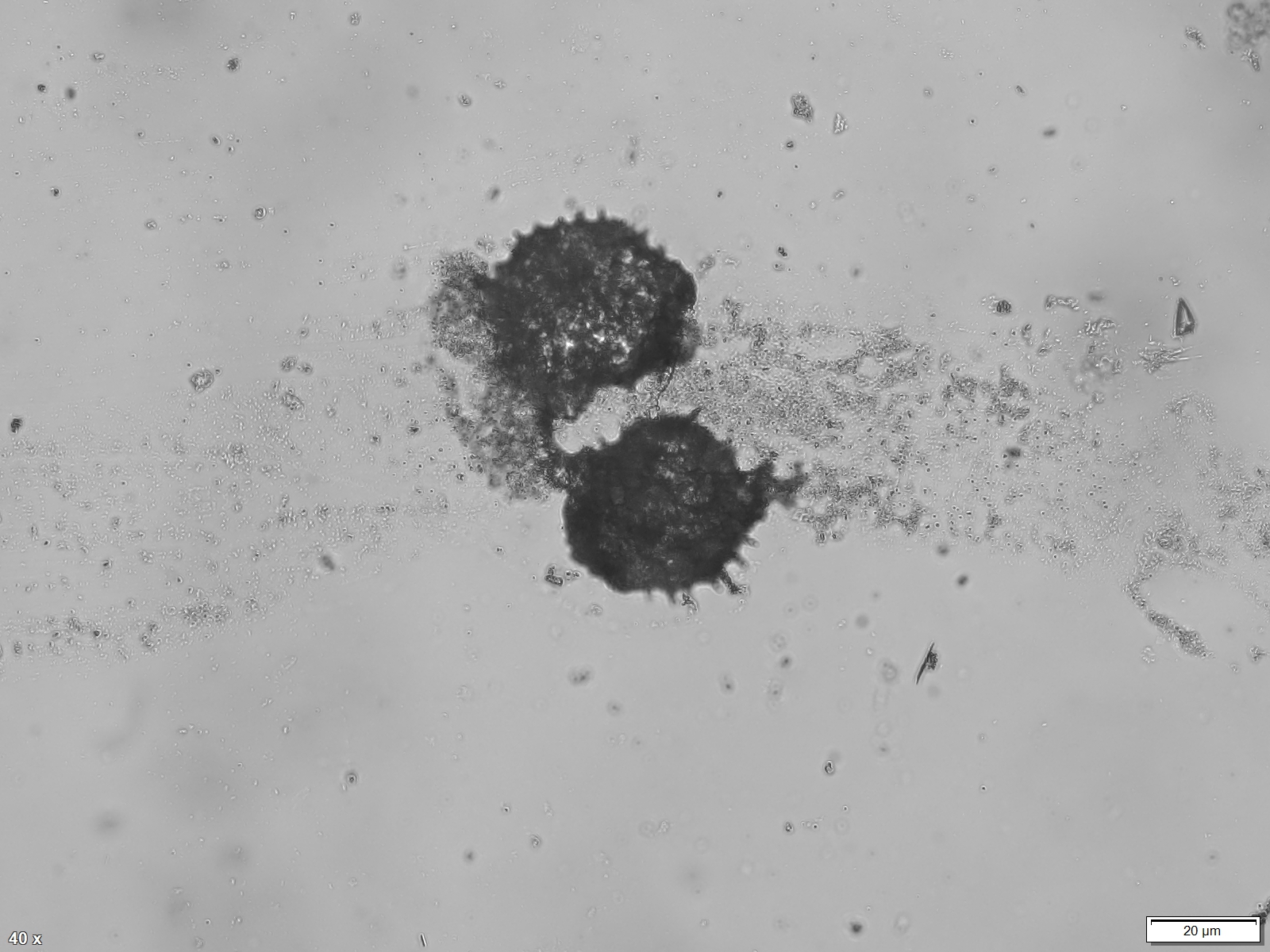

Supplement: S1 File — (ZIP) [file pone.0230519.s001.zip › Image_2009.jpg]

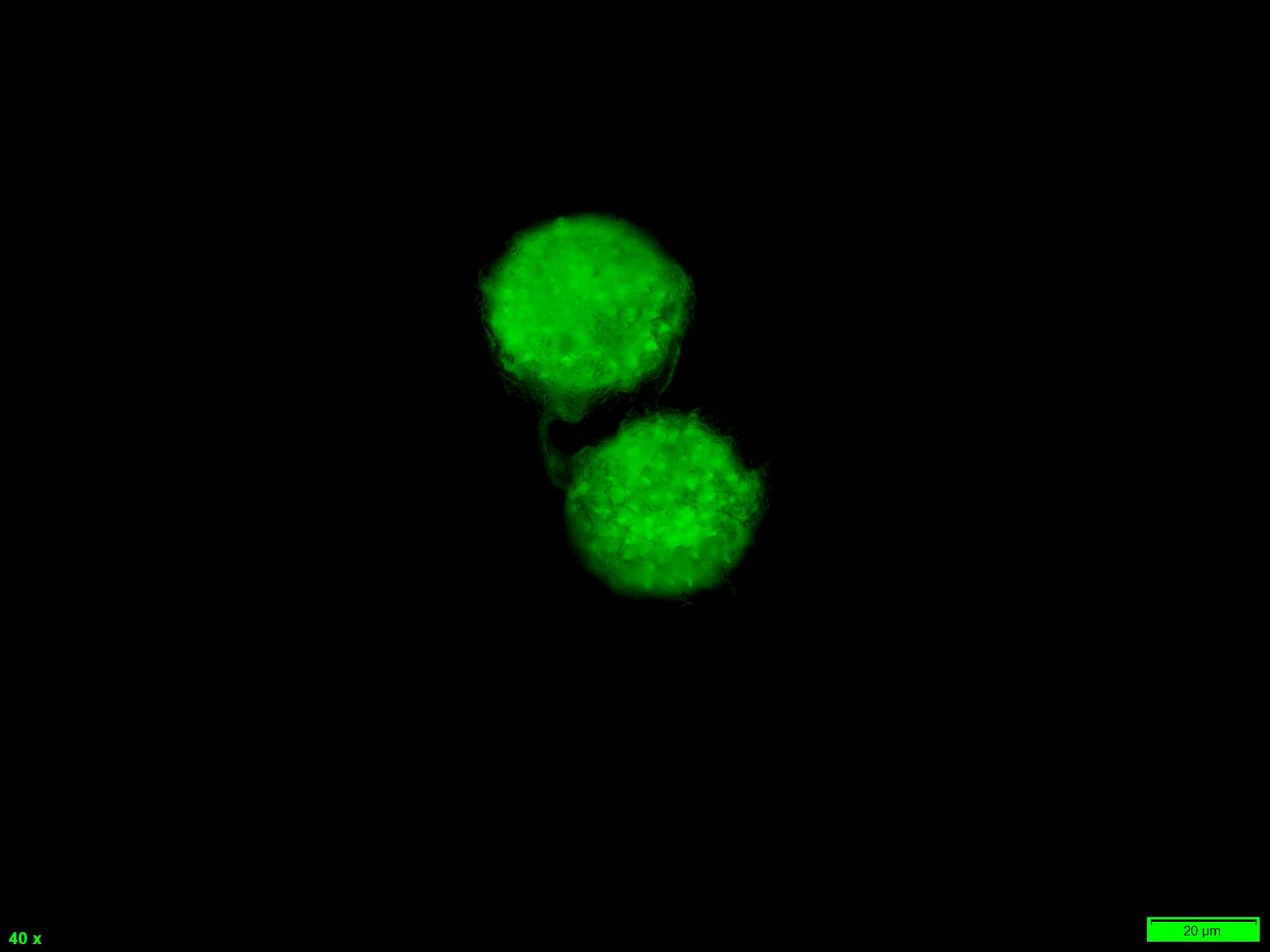

Supplement: S1 File — (ZIP) [file pone.0230519.s001.zip › Image_2011.jpg]

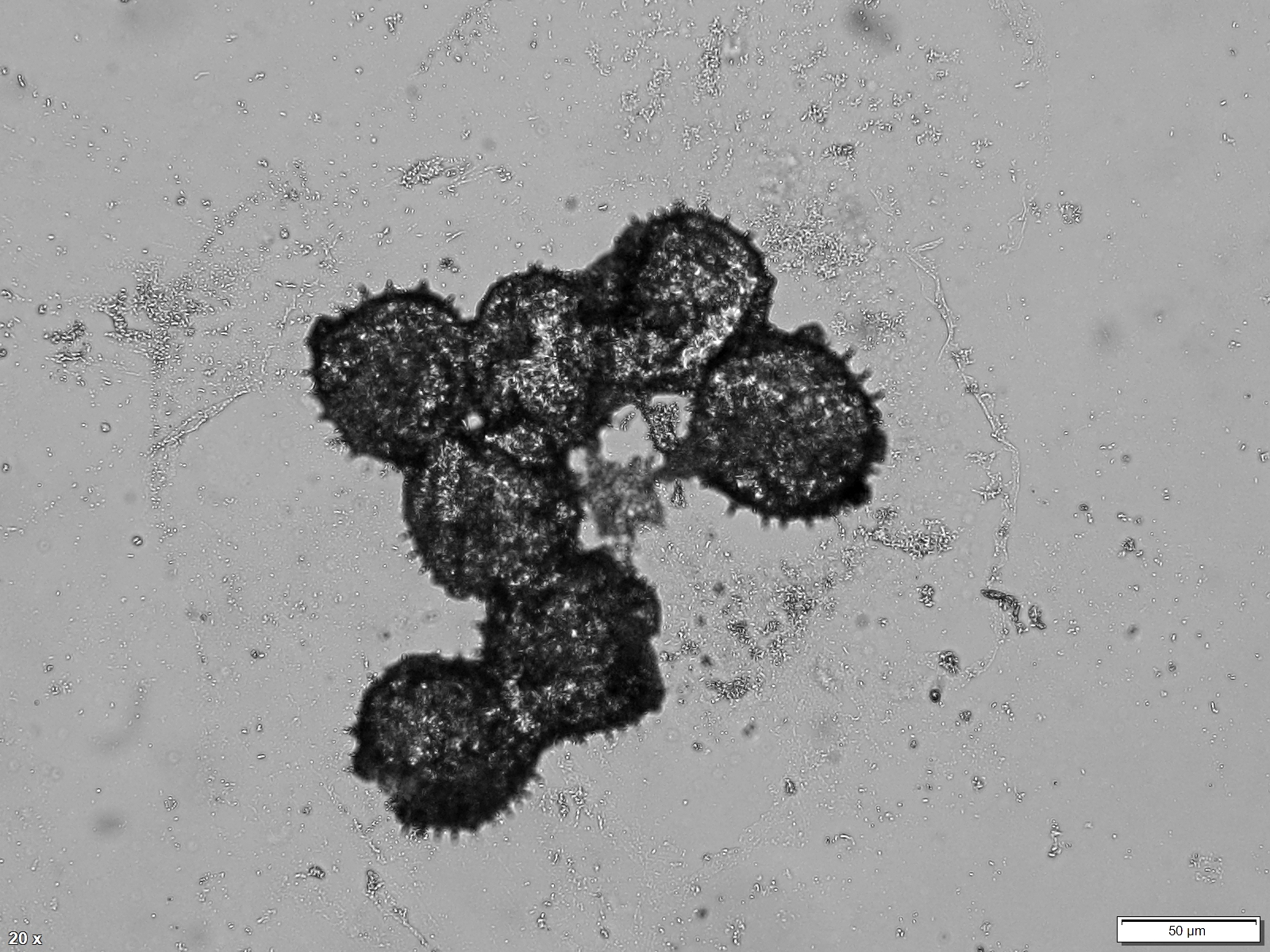

Supplement: S1 File — (ZIP) [file pone.0230519.s001.zip › Image_2016.jpg]

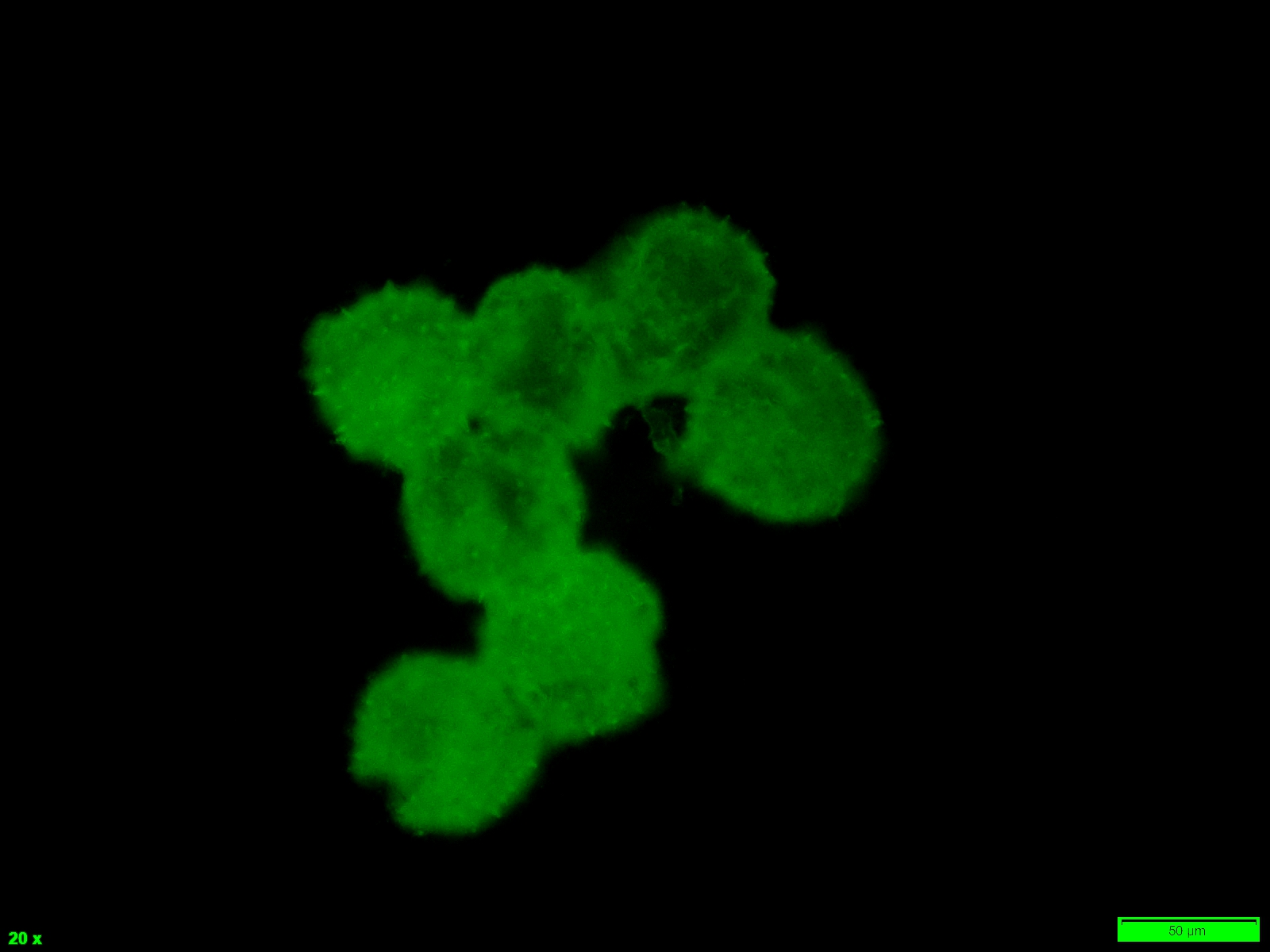

Supplement: S1 File — (ZIP) [file pone.0230519.s001.zip › Image_2018.jpg]

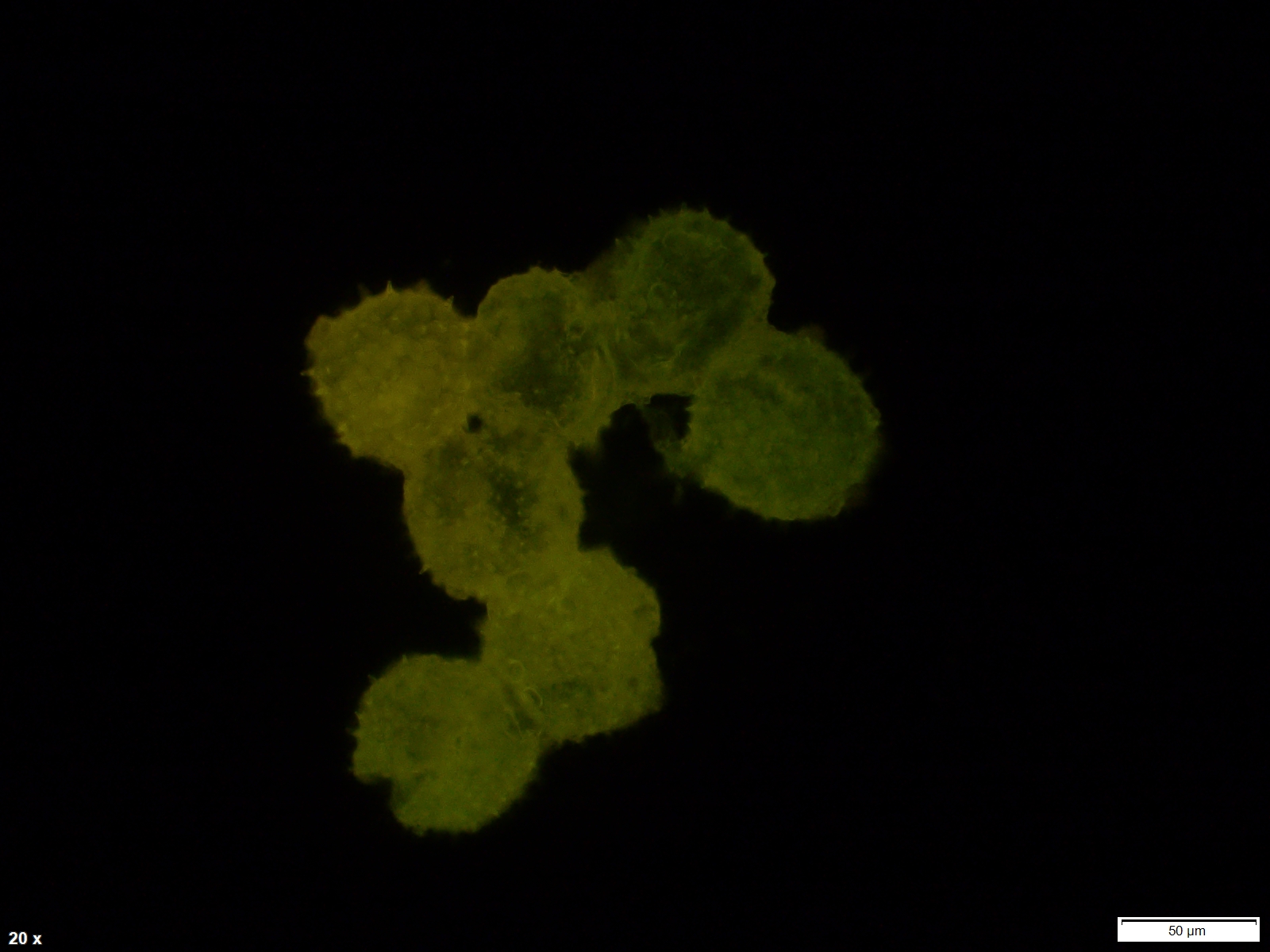

Supplement: S1 File — (ZIP) [file pone.0230519.s001.zip › Image_2019.jpg]

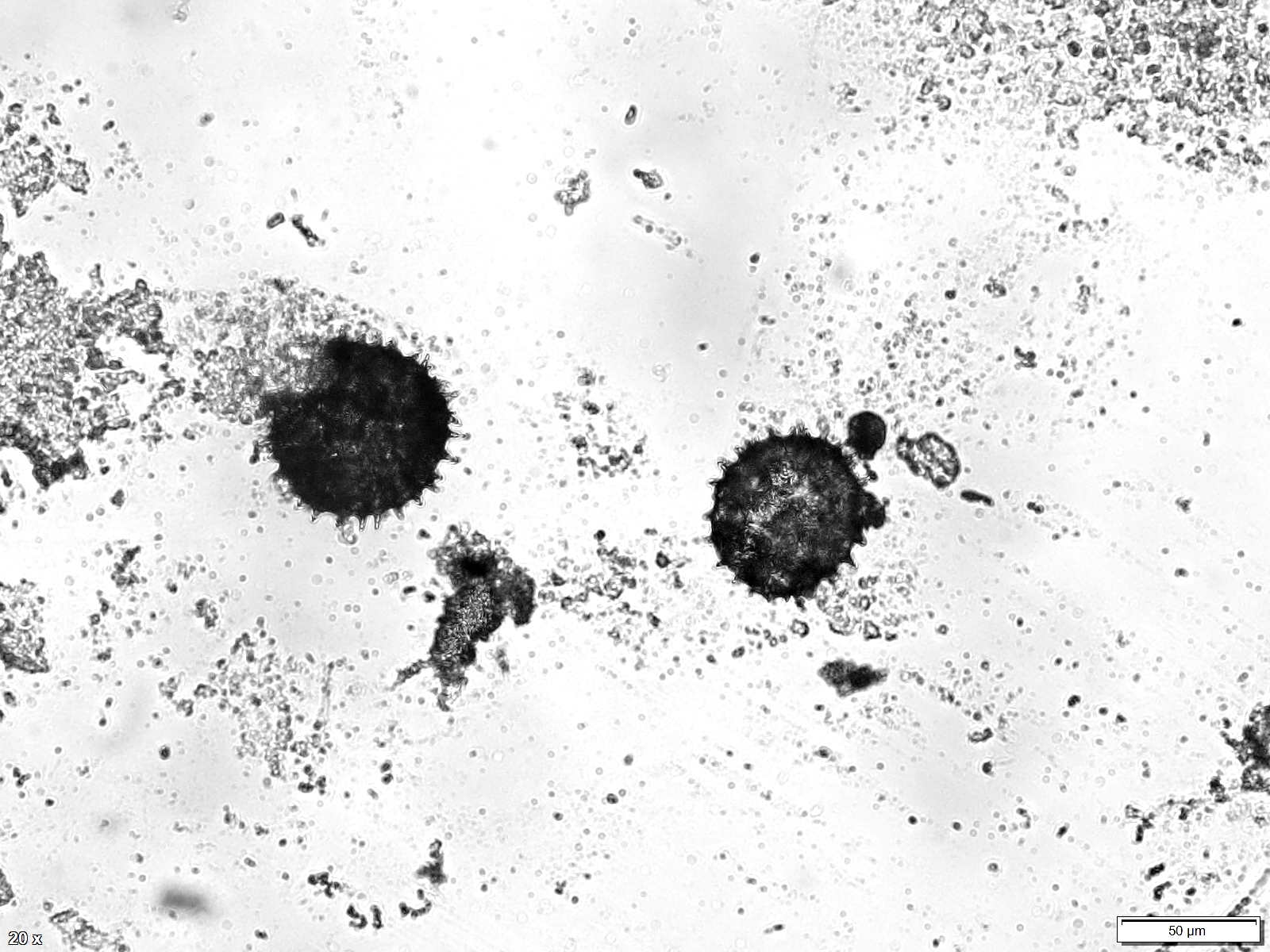

Supplement: S1 File — (ZIP) [file pone.0230519.s001.zip › Image_2021.jpg]

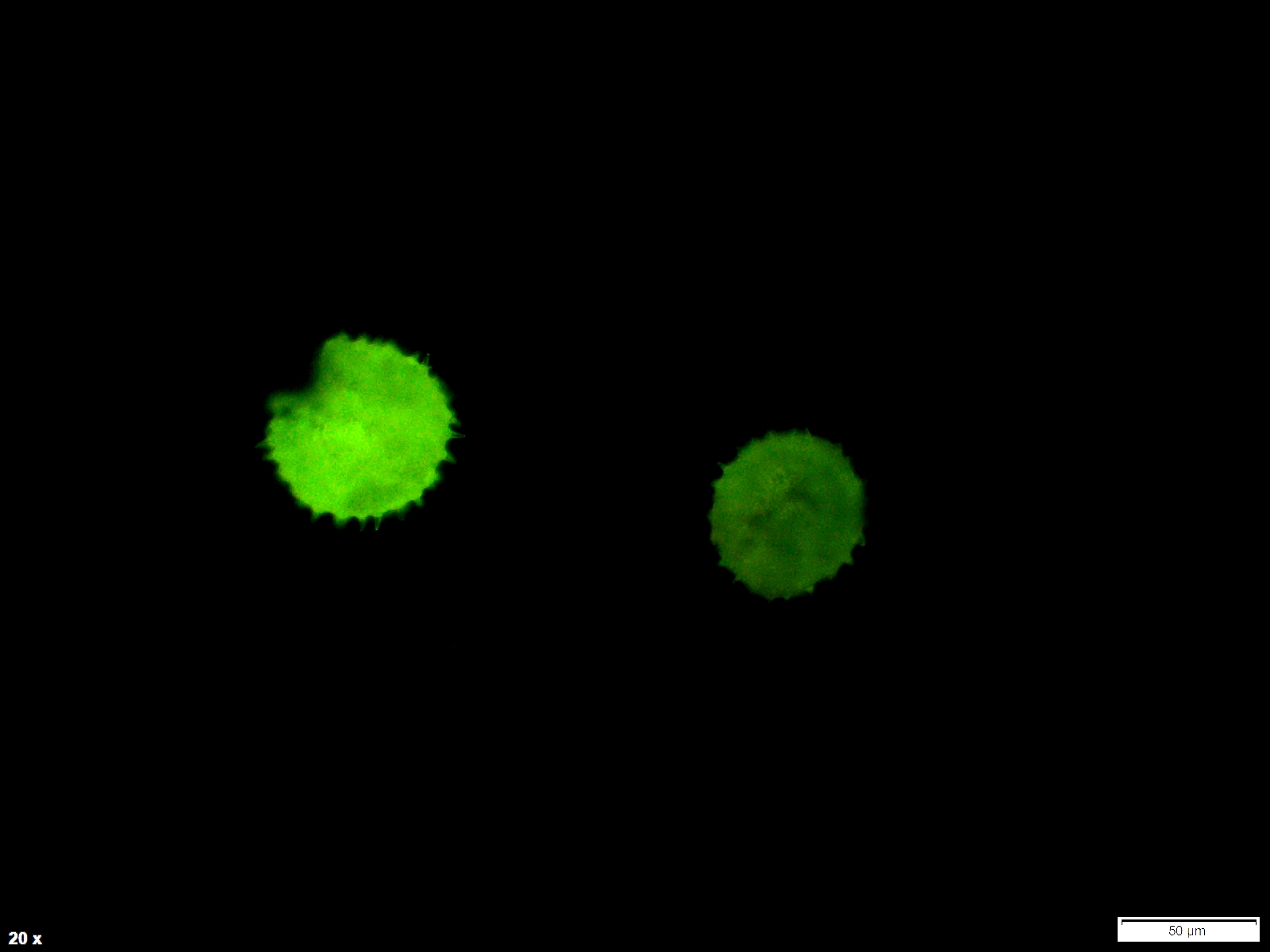

Supplement: S1 File — (ZIP) [file pone.0230519.s001.zip › Image_2022.jpg]
